# Multi‐omics analysis of gut‐organ axes reveals the high‐altitude adaptation in Tibetan chicken

**DOI:** 10.1002/imo2.70038

**Published:** 2025-06-29

**Authors:** Tao Zeng, Tiantian Gu, Yongqing Cao, Yong Tian, Jianmei Yin, Peishi Feng, Hanxue Sun, Jindong Ren, Xueying Ma, Zelong Zhao, Guohui Li, Li Chen, Wenwu Xu, Qian Xue, Wei Han, Lizhi Lu

**Affiliations:** ^1^ State Key Laboratory for Quality and Safety of Agro‐Products Key Laboratory of Livestock and Poultry Resources (Poultry) Evaluation and Utilization, Ministry of Agriculture and Rural Affairs, Institute of Animal Husbandry and Veterinary Science, Zhejiang Academy of Agricultural Sciences Hangzhou China; ^2^ Zhejiang Key Laboratory of Livestock and Poultry Biotech Breeding, Zhejiang Provincial Engineering Research Center for Poultry Breeding Industry and Green Farming Technology Hangzhou China; ^3^ National Chickens Genetic Resources, Jiangsu Institute of Poultry Science Yangzhou China; ^4^ Technology Innovation Co., Ltd., Jiangsu Institute of Poultry Science Yangzhou China; ^5^ Zhejiang University of Technology Hangzhou China; ^6^ Institute of Animal Husbandry and Veterinary Medicine Tibet Academy Agricultural and Animal Husbandry Sciences Lhasa China; ^7^ Shanghai BIOZERON Biotechnology Co., Ltd. Shanghai China

**Keywords:** environmental adaptation, fatty acid metabolism, gut microbiota, gut‐organ axis, immune activity, poultry

## Abstract

The Qinghai‐Tibet Plateau is an extreme ecosystem subject to special climatic conditions that require unique adaptations for its inhabiting organisms. In addition to genetic characteristics, the gut microbiota of animals can regulate the environmental adaptation of their hosts through various gut–organ axes. We performed a multi‐omics analysis on six Chinese chicken populations based on geographical differences in altitude: one high‐altitude Tibetan chicken population, one transitional Tibetan chicken population relocated from high to low altitude, and four low‐altitude populations. We found significant differences in gut microbiota among these different chicken populations, which were governed by variations in habitat species pools and turnover, indicating a more complex and stochastically dominated gut microbiota with higher functional redundancy in the Tibetan chicken population under the plateau environment. Furthermore, Tibetan chickens had a more effective fatty acid degradation capacity, corresponding to the hypoxic environment. In contrast, chickens living in lowland environments showed stronger immune system responses against health threats. These environmental adaptation strategies are regulated by core gut microbe taxa of the phylum Firmicutes. Thus, our findings demonstrate the roles of breed and habitat in gut microbiota composition of chickens and clarify their adaptation strategies to environmental changes via microbiota‐driven gut–organ axes.

## Highlights

- Tibetan chickens exhibit distinct gut microbial communities shaped by high‐altitude conditions with higher functional redundancy and stochastic dominance.
- Tibetan chickens had a more effective fatty acid degradation capacity, corresponding to the hypoxic environment.
- Firmicutes as core regulators of host environmental adaptation.

## Graphical abstract

The Qinghai‐Tibet Plateau is an extreme ecosystem subject to special climatic conditions that require unique adaptations for its inhabiting organisms. In addition to genetic characteristics, the gut microbiota of animals can regulate the environmental adaptation of their hosts through various gut–organ axes. We performed a multi‐omics analysis on six Chinese chicken populations: one high‐altitude Tibetan chicken population, one transitional Tibetan chicken population relocated from high to low altitude, and four low‐altitude populations. We found that the Tibetan chicken population under the plateau environment indicated a more complex and stochastically dominated gut microbiota with higher functional redundancy. Furthermore, Tibetan chickens had a more effective fatty acid degradation capacity, corresponding to the hypoxic environment. In contrast, chickens living in lowland environments showed stronger immune system responses against health threats, mainly regulated by the phylum Firmicutes. Thus, our findings clarify their adaptation strategies to environmental changes via microbiota‐driven gut–organ axes.

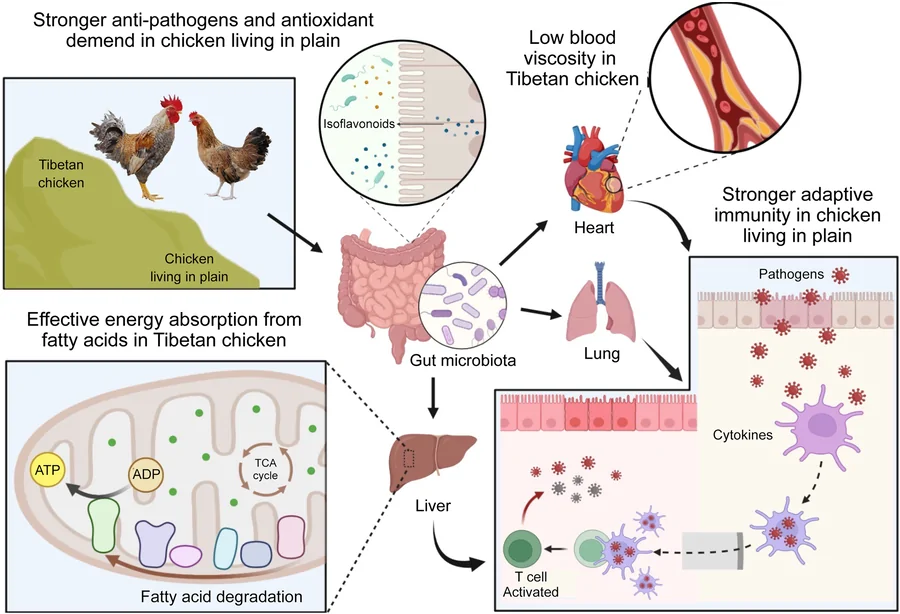

## 1 INTRODUCTION

The Qinghai‐Tibet Plateau has been described as the “third pole of the earth” because of its extreme environmental conditions, such as low oxygen level, high UV radiation, and cold temperature, which are challenging for many species [1]. Tibetans, as well as Tibetan wild and domesticated animals living on the plateau, have adapted to the hypoxic environment and have developed unique lifestyles, dietary habits, and genetic characteristics [2, 3, 4, 5, 6]. Studies have shown that environmental factors such as low oxygen and UV radiation could enhance immune cell function and adaptive responses based on high altitude adaptation [7, 8]. Another key aspect of animal adaptation to hypoxic environments is gut microbiota [9], which is closely associated with host health and environmental adaptation [10, 11]. Several studies have found significant differences between the intestinal bacteria of high and low‐altitude humans or animals [12, 13, 14], but few have elucidated the underlying adaptive mechanism of the hypoxic environment in mediating host adaptations. Further research studies found significant altitude‐correlated differences in the diversity of gut microbiota in humans, Tibetan pigs (*Sus domesticus*), Tibetan wild asses (*Equus kiang*), and yaks (*Bos grunniens*) [15, 16, 17, 18], suggesting that they may contribute to their hosts' adaptation to the prevailing conditions.

The Tibetan chicken (*Gallus gallus*) is a unique breed native to the Qinghai‐Tibet Plateau that has acquired distinctive adaptations to the high‐altitude environment over thousands of years [19]. In contrast to chicken breeds from lowland plains, Tibetan chickens have a higher venous CO_2_ partial pressure, higher blood hemoglobin concentration, and hemoglobin with stronger oxygen affinity [20]. These unique characteristics make this breed an interesting case study for understanding the adaptations of gut microbiota to the Tibetan plateau's hypoxic environment. It has been reported that differences in the cecal microbiota of Tibetan chickens from five geographic regions were correlated with their different environments [21]. Another study found that the composition and diversity of gut microbes in Tibetan chickens changed after they were introduced to lower altitudes [13]. However, the assembly mechanisms of gut microbiota in Tibetan chickens regarding low‐altitude adaptation are still missing. Multiple studies have uncovered the importance of gut microbiota on host physiology via various “gut–organ” axes, which regulate the gene expression of organs through intestinal metabolites [22, 23]. In addition, the interactions of gut microbiota and host gene expression in Tibetan chickens associated with their adaptation to the plateau's hypoxic environment remain unclear.

**FIGURE 1:**
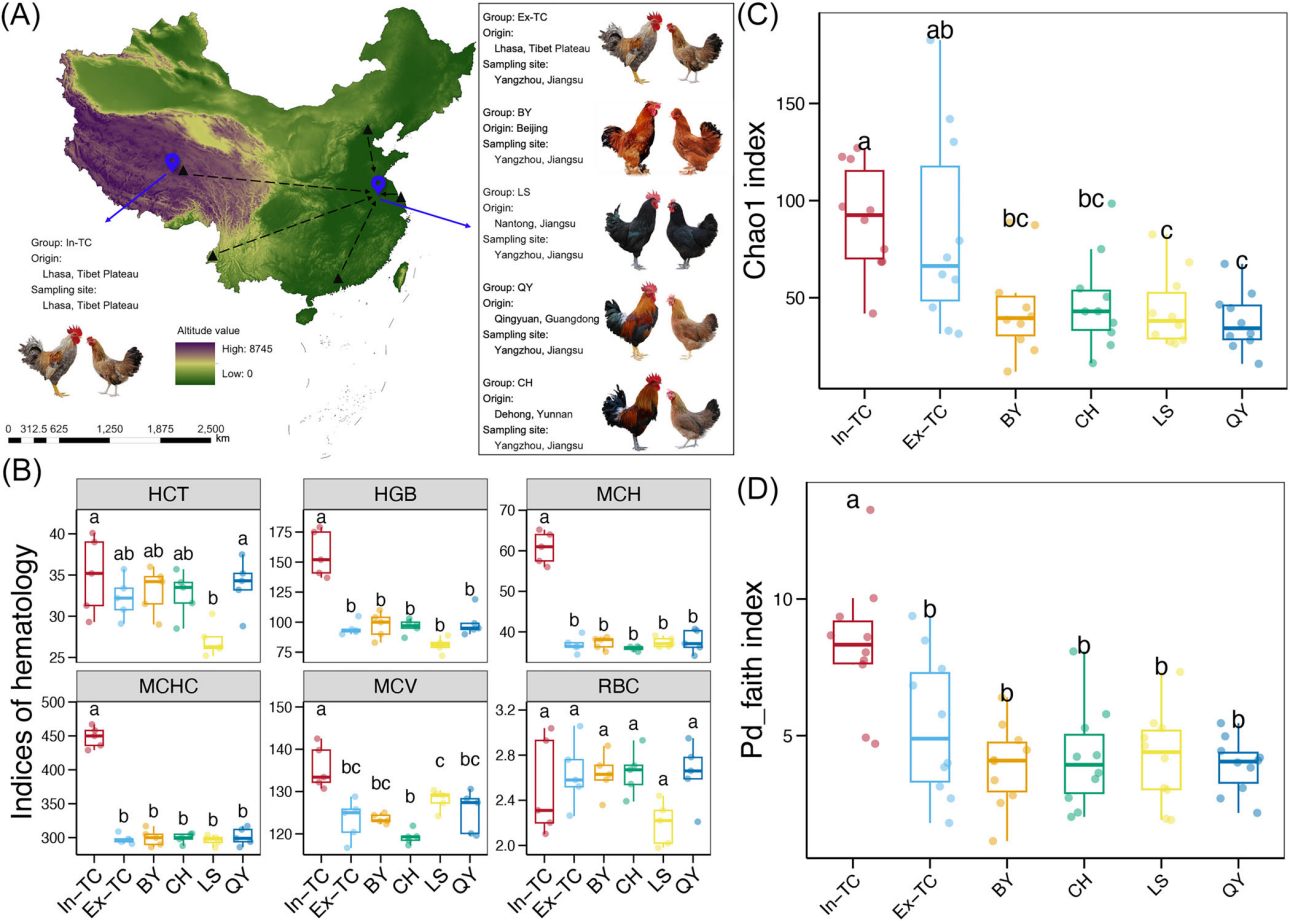
The differences in blood physiological indices and gut microbiota diversity among chicken populations. (A) Schematic diagram of sample collection. (B) Differences in blood physiological indices among chicken populations. (C, D) Differences in the Chao1 and Pd_faith indices, respectively, of gut microbiota among different chicken populations. Lowercase letters above the boxes represent significant differences (Dunn's test, *p* < 0.05) between groups. In‐TC, Tibetan chicken on the plateau; Ex‐TC, Tibetan chicken on the plain; BY, Beijing You chicken; CH, Chahua chicken; LS, Langshan chicken; QY, Qingyuan Ma chicken; HCT, hematocrit; HGB, hemoglobin; MCH, mean erythrocyte hemoglobin content; MCHC, mean corpuscular‐hemoglobin concentration; MCV, mean corpuscular volume; RBC, red blood cells.

In this study, we selected six populations from five representative Chinese chicken breeds across different altitudes: one high‐altitude Tibetan chicken population (In‐TC, Tibetan Plateau), one transitional Tibetan chicken population relocated from high to low altitude (Ex‐TC) after nearly 20 years of ex situ in vivo conservation, and four low‐altitude populations (BY, Beijing You chicken; LS, Langshan chicken; QY, Qinyuan Ma chicken; CH, Chahua chicken) from various regions (Figure 1A). We compared the gut microbiota of these populations through high‐throughput sequencing to analyze diversity, composition, and assembly mechanisms. Additionally, we utilized nontargeted metabolomics to identify metabolites linked to host adaptation and RNA‐seq to profile gene expression across different organs. This multi‐omics approach offers new insights into how Tibetan chickens adapt to the hypoxic plateau environment through gut‐organ interactions.

## 2 RESULTS

### Differences in blood physiological indices

We measured six blood physiological indices among the different chicken populations (Figure 1A), including hematocrit (HCT), hemoglobin (HGB), mean erythrocyte hemoglobin content (MCH), mean corpuscular hemoglobin concentration (MCHC), mean corpuscular volume (MCV), and red blood cells (RBC). The concentrations of HGB, MCH, MCHC, and MCV in In‐TC were much higher than those in plains populations, including the Ex‐TC group (Tukey's HSD test, *p* < 0.05), while no significant difference was observed among plains populations (Figure 1B). There were no significant differences in the concentration of HCT and RBC between In‐TC and the other five groups (Figure 1B).

### Gut microbiota sequencing

To investigate the effect of gut microbiota composition regarding high‐altitude adaptation, we sequenced a total of 60 cecum content samples from six chicken populations (10 per population) and obtained 704,194 high‐quality reads (average 12,986 per sample), which clustered to 3619 amplicon sequence variants (ASVs). All ASVs were successfully annotated for bacterial phyla with ~90% taxonomic annotation at the genus level, but only 34.41% of them could be assigned to species (Figure S1). We identified 15 phyla, 20 classes, 39 orders, 65 families, 118 genera, and 95 species, with clearly higher numbers in the In‐TC group compared to the lowland populations (Table S1). The rarefaction curves of all six populations tended to be horizontal (Figure S2), indicating that the sequencing depth reflected the complete gut microbiota. Significantly higher Chao1 indices were found in In‐TC samples compared to plains populations (Dunn's test, *p* < 0.05), while no difference was observed among plains populations (Figure 1C). In addition, the Chao1 index showed obvious differences between the plateau (In‐TC) and plains populations (Ex‐TC) of Tibetan chicken (Figure 1C). The Pd_faith index in In‐TC samples was significantly higher than that in plains populations, including the Ex‐TC group (Dunn's test, *p* < 0.05, Figure 1D). In contrast, no significant variation was found in Shannon and Pielou_J index values between any two populations (Dunn's test, *p* > 0.05, Figure S3). These results suggest a higher richness and phylogenetic diversity in gut microbiota of chicken populations from the plateau compared to those from lowland plains.

### Diversity variations of gut microbiota

**FIGURE 2:**
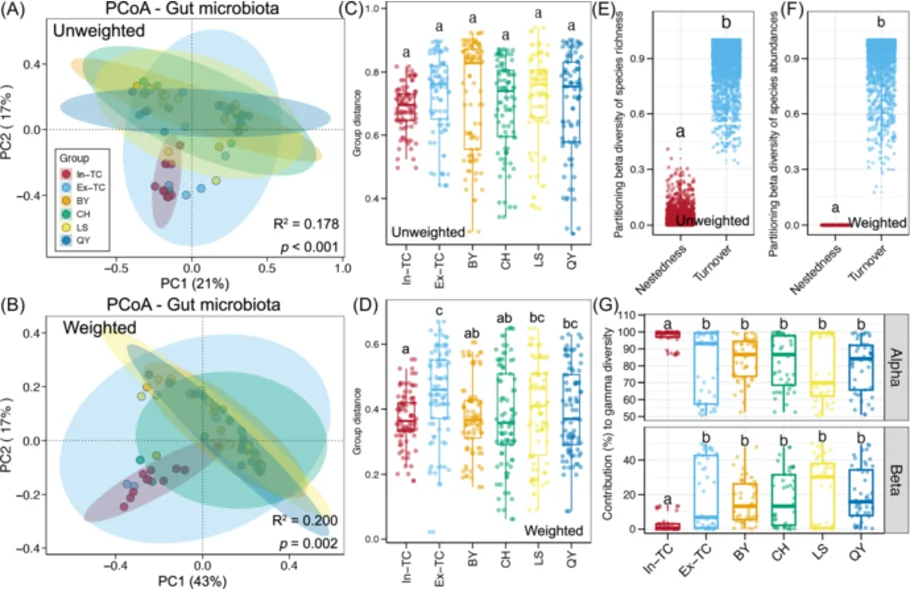
Changes in gut microbial community among chicken populations. (A, B) Principal coordinate analysis (PCoA) and Adonis test of chicken cecal microbiota based on unweighted and weighted Unifrac distances, respectively. (C, D) Differences in the intra‐variations of gut microbiota among different chicken populations according to Unweighted and Weighted Unifrac distances, respectively. (E, F) The relative importance of nestedness and turnover on β diversity of chicken cecal microbiota based on unweighted and weighted Unifrac distances, respectively. (G) Contributions of α and β diversity to the variations in γ diversity of gut microbiota among different chicken populations. Lowercase letters above the boxes represent significant differences (Tukey's HSD, *p* < 0.05) between groups.

Based on the results of α diversity, we applied Unifrac distances to assess the β diversity of chicken cecal microbiota because it accounts for phylogeny [24]. We calculated both unweighted and weighted distances, which characterize the effects of the local species pool and relative species abundance, respectively [25]. Principal coordinate analysis (PCoA) based on the unweighted Unifrac distance showed a distinct separation of the In‐TC group from the plains populations (including Ex‐TC) along the PC2 axis, explaining 17% of the total variation (Figure 2A). The Adonis test confirmed the significant explanatory power of population identity on gut microbiota composition (*R* ^2^ = 0.178, *p* < 0.05). According to the weighted Unifrac distance, the In‐TC group also clustered separately from other groups (Adonis test, *R* ^2^ = 0.200, *p* < 0.05, Figure 2B). We found no significant differences in intra‐group variation among populations based on the unweighted Unifrac distance (Figure 2C). However, variation was significantly smaller in the In‐TC group than that in the plain populations (Figure 2D). These results indicate that both the local species pool and relative species abundance have effects on the variation in gut microbiota among the different chicken populations. The local species pool appears to contribute more to the variations between groups, whereas relative species abundance is more influential regarding variations within a single population.

The β diversity of communities can be divided into nestedness‐ and turnover‐driven forms [26]. The former implies that changes in the community derive from the emergence or loss of species, while the latter is driven by the replacement of species between different communities [27]. In both unweighted and weighted Unifrac distance analyses, the contributions of turnover were more important than nestedness (Student's *t*‐test, *p* < 0.05, Figure 2E,F). However, we observed no differences among different populations irrespective of nestedness or turnover, neither unweighted nor weighted Unifrac distances (Figure S4). We further quantified the relative contributions of α and β diversity to γ diversity (Figure 2G). The α diversity of the In‐TC population contributed about 97% of the variation in the gut microbiota at this level, which was significantly more than that in the plains populations (averaging 75.3%–84.0%). In contrast, the contributions of β diversity showed the opposite trend, with the plains populations outweighing the In‐TC group (Figure 2G). These findings suggest a fundamental role of host population and environment in governing succession in gut microbiota of different chicken populations.

### Differences in gut microbiota composition

According to our taxonomic analyses, Firmicutes was the most abundant bacterial phylum in chicken cecal microbiota (58.2%), followed by Proteobacteria (17.3%), Bacteroidota (16.9%), and Actinobacteriota (4.0%) (Figure S5). The relative abundances of Proteobacteria and Actinobacteriota were significantly lower in the In‐TC samples compared to those in the plains population samples (including the Ex‐TC group) (Tukey's HSD test, *p* < 0.05, Figure S6). In contrast, several bacterial phyla with considerable abundances in the In‐TC group (such as Defferribacterota, Desulfobacterota, Fusobacteriota, and Verrucomicrobiota) were almost absent in the lowland populations (Figure S6). Among bacterial genera, *Lactobacillus* was the most dominant (12.3%), with other genera including *Ligilactobacillus*, *Escherichia‐Shigella*, and *Limosilactobacillus* that present at proportions of >5% (Figure S7). *Bacteroides*, *Anaerosporobacter*, *Desulfovibrio*, and *Phascolarctobacterium* were found to be specifically enriched in gut microbiota of the In‐TC group (Figure S8).

**FIGURE 3:**
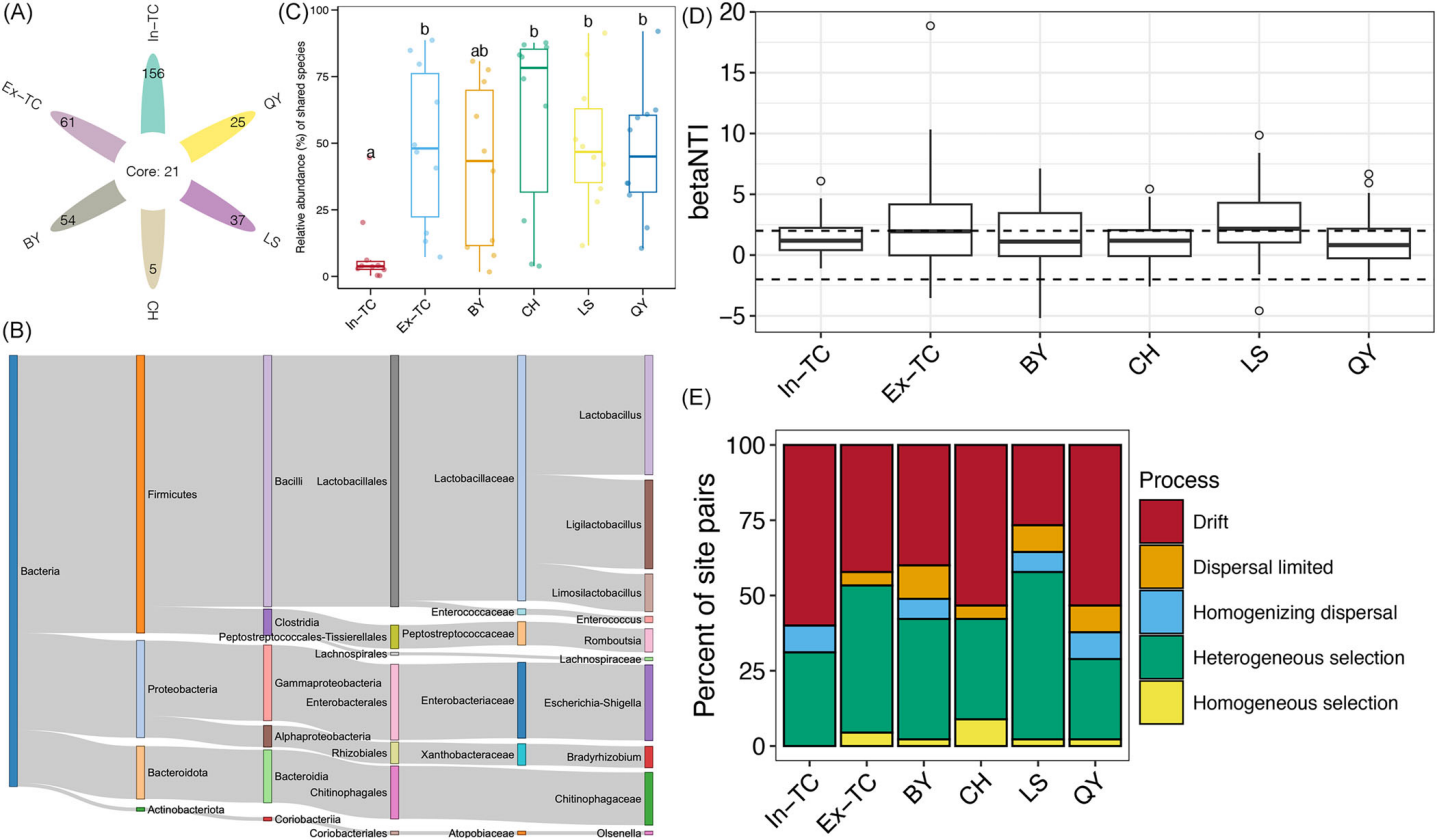
The potential mechanisms of gut microbiota regulation in high‐altitude adaptation. (A) Venn diagram for identifying shared amplicon sequence variants (ASVs) among the six studied chicken populations. (B) Sankey diagram showing the taxonomy of shared ASVs. (C) Differences in the summed abundances of shared ASVs among different chicken populations. Lowercase letters above the boxes represent significant differences (Tukey's HSD, *p* < 0.05) between groups. (D) Variations in beta‐nearest taxon index (betaNTI) among the gut microbiota of different chicken populations. (E) Contributions of different ecological processes to gut microbiota assembly in different chicken populations.

Only 21 bacterial ASVs were shared among the studied chicken populations (Figure 3A), which belonged to four phyla and ten genera and showed a composition similar to that of the complete microbiota (Figure 3B). The total abundance of these shared ASVs was only 10% in the In‐TC group compared to that of ~50%–75% in the plains populations (Figure 3C). These outcomes further validate the finding of the β‐diversity analysis that differences in the local species pool were drivers of the interpopulation variations in chicken cecal microbiota.

### Assembly mechanisms of gut microbiota

We used the betaNTI index to evaluate the gut microbiota assembly in different chicken populations. Median values of betaNTI were between −2 and 2, except for the LS group, which had a value slightly above 2 (Figure 3D). While these values indicate that stochastic processes governed the assembly of a majority of the microbiota, there were still population‐specific differences. Drift‐dominated stochastic processes, while heterogeneous selection dominated deterministic processes for microbiota assembly in the In‐TC group (Figure 3E). While this was also true for the plains populations, with varied specific contributions. The relative importance of drift was lower in the plains populations (26.67%–53.33%) than that in the In‐TC group (60%), but there emerged an additional stochastic process of dispersal limitation (4.44%–11.11%) in the plains populations (Figure 3E). For deterministic processes, the contribution of heterogeneous selection was similar or greater in the plains populations (26.67%–55.56%) than that in the In‐TC group (31.11%), and homogeneous selection also occurred (4.44–11.11%) in the plains populations (Figure 3E).

### Effects of habitat on gut microbiota of Tibetan chicken

To enhance our understanding of the characteristics of Tibetan chicken, we further compared the gut microbiota between the In‐TC and Ex‐TC groups, which had the same ancestors but had been raised in different habitats for nearly 20 years. We observed significant intergroup differences in evolutionary diversity and composition of gut microbiota (Figure 1D, Figure 2). A total of 110 ASVs were found to be shared between these two groups (Figure S9), which was substantially higher than the proportion shared among all six studied populations (Figure 3A). However, the total abundance of these shared ASVs was more limited in the gut microbiota of In‐TC samples (~35%) than that in the Ex‐TC samples (~75%) (Figure S10). *Bacteroides* were significantly enriched in the In‐TC group, while *Limosilactobacillus* and *Lactobacillus* were more abundant in Ex‐TC individuals (Figure S11). The predicted functions of microbiota indicated a higher abundance of functions involved in cellular processes in the In‐TC group, and more abundant functions related to human diseases in the Ex‐TC group (Figure S12).

**FIGURE 4:**
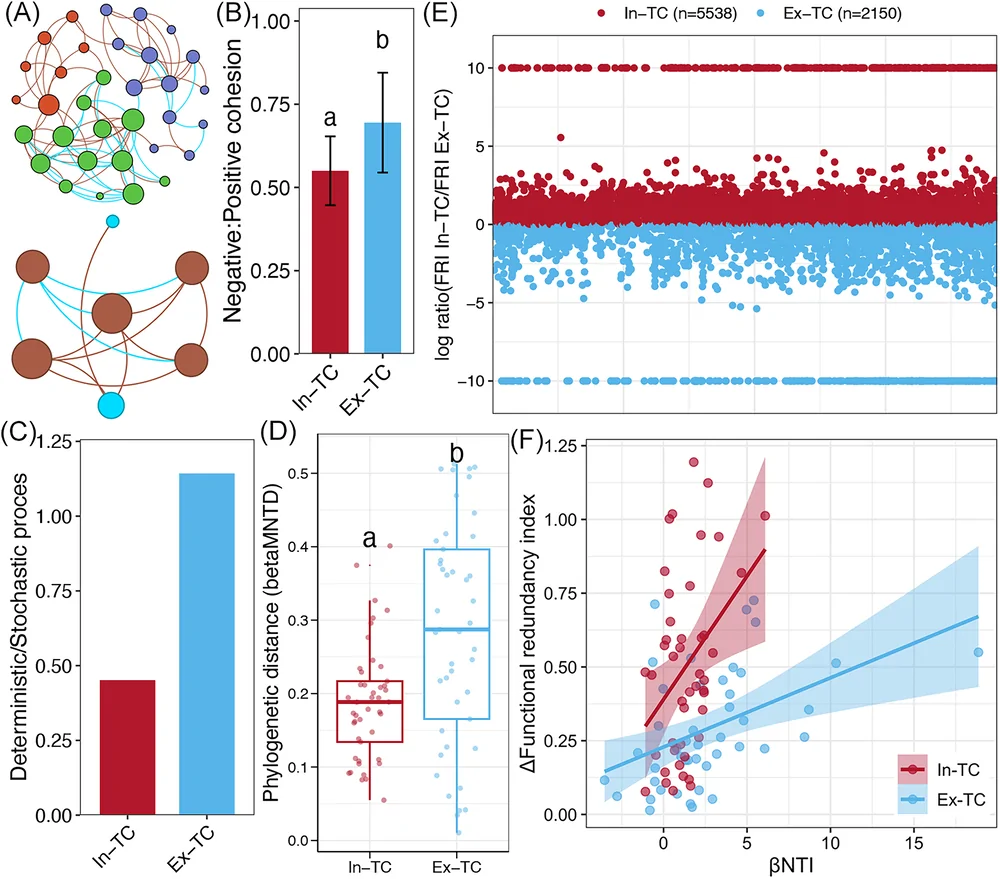
Effects of habitat on gut microbiota of Tibetan chicken. (A) Co‐occurrence networks of gut microbiota in the plateau (In‐TC) and plains populations (Ex‐TC) of Tibetan chicken. Modules are labeled in different colors in the respective network. Red line indicates a positive correlation, while blue line indicates a negative correlation. (B) Differences in N:P cohesion between gut microbiota in the In‐TC and Ex‐TC groups. (C) Ratio of relative contributions of deterministic and stochastic processes to the assembly of gut microbiota in the In‐TC and Ex‐TC groups. (D) Differences in phylogenetic distance between gut microbiota in the In‐TC and Ex‐TC groups. (E) Log ratio of functional redundancy index (FRI) between gut microbiota in the In‐TC and Ex‐TC groups. (F) Linear regression between betaNTI and FRI for gut microbiota in the In‐TC and Ex‐TC groups. Lowercase letters above the boxes represent significant differences (Student's *t*‐test, *p* < 0.05) between groups.

We constructed co‐occurrence networks of gut microbiota from the In‐TC and Ex‐TC groups (Figure 4A). The In‐TC group showed a substantially more complex network based on the comparison of topological parameters (Table S2). In contrast, we detected significantly lower Negative: Positive (N:P) cohesion in the In‐TC network (Student's *t*‐test, *p* < 0.05, Figure 4B), indicating a low stability of gut microbiota. Based on the results of the null model analysis, the gut microbiota assembly appeared to have changed from a stochastic‐driven state in the In‐TC group to a deterministic‐driven state in the Ex‐TC population (Figure 4C). Comparison of the betaMNTD between the In‐TC and Ex‐TC groups revealed a higher value among samples of the Ex‐TC population than those of the In‐TC population (Figure 4D). Function prediction indicated that 5,538 function terms were associated with the gut microbiota in the In‐TC group, implying a greater degree of redundancy among functions, while less than half (2150) were found in the Ex‐TC group (Figure 4E). We further found significant positive correlations between the betaNTI and functional redundancy index (FRI) in both groups (linear regression, *p* < 0.05, Figure 4F). The slope for correlation of betaNTI and FRI was higher in the In‐TC group than that in the Ex‐TC group, implying a closer correlation between gut microbiota assembly and their functional redundancy in the In‐TC population. Taken together, individuals of the In‐TC population had a predominantly stochastic gut microbiota with greater complexity, lower stability, greater functional redundancy, and narrower phylogenetic distribution compared to the Ex‐TC population.

### Relationships between gut microbiota and their metabolites

**FIGURE 5:**
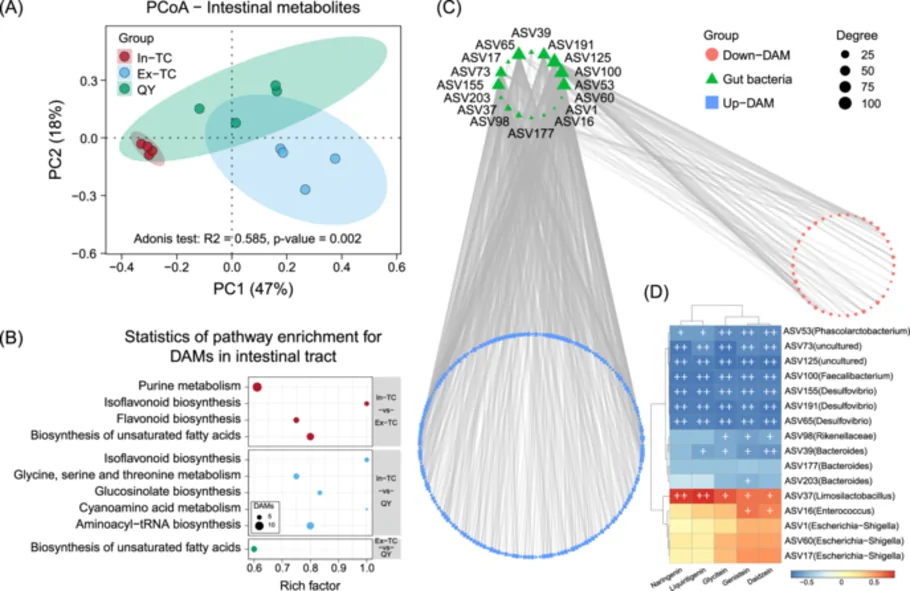
Correlations of gut microbial communities with their metabolites. (A) Principal coordinate analysis (PCoA) showing the differences in intestinal metabolome among In‐TC, Ex‐TC, and QY populations. (B) Kyoto Encyclopedia of Genes and Genomes (KEGG) enrichment analyses based on differentially abundant metabolites (DAMs) identified from different comparisons. (C) Correlation network of gut microbiota and potential key DAMs. (D) Heatmap showing correlations between key gut bacterial ASVs and DAMs involved in the isoflavonoid biosynthesis pathway. “++” and “+” represent *p*‐values <0.01 and <0.05, respectively. ASVs, amplicon sequence variants.

We investigated the gut metabolome of the In‐TC, Ex‐TC, and QY populations to explore the potential mechanisms behind the plateau adaptability of the In‐TC population. Based on ion current profiles, we identified approximately 1500 metabolites from the chicken intestinal tract. PCoA showed separate clusters of different populations with obviously smaller intra‐group variations for the In‐TC group (Figure 5A). A total of 389, 406, and 181 metabolites were identified as differentially abundant metabolites (DAMs) in comparisons of In‐TC versus Ex‐TC, In‐TC versus QY, and Ex‐TC versus QY, respectively (Figure S13). The number of DAMs between the native Tibetan chicken and either of the plains populations was higher than that for the two plains populations, which was mainly contributed by the upregulated DAMs (286 and 324 vs. 103, Figure S13).

We further performed metabolome enrichment analyses, which showed that DAMs in In‐TC versus Ex‐TC were significantly enriched in pathways of purine metabolism, (iso)flavonoid biosynthesis, and biosynthesis of unsaturated fatty acids (Figure 5B). We also found that several pathways were significantly enriched in In‐TC versus QY, including isoflavonoid biosynthesis, glycine, serine, and threonine metabolism, glucosinolate biosynthesis, cyanoamino acid metabolism, and aminoacyl‐tRNA biosynthesis (Figure 5B). In contrast, only one pathway (biosynthesis of unsaturated fatty acids) was enriched in Ex‐TC versus QY. We proposed that enriched pathways specifically present in the plateau populations could be important for the plateau adaptability of the Tibetan chicken. Thus, only one pathway, isoflavonoid biosynthesis, met this requirement (Figure 5B). Consistently, five DAMs identified in this pathway (liquiritigenin, daidzein, glycitein, naringenine, and genistein) were all downregulated in the In‐TC group compared to the plains populations (Figure S14).

In all three comparisons, 213 upregulated and 44 downregulated DAMs were identified as potential key metabolites for plateau adaptability, based on the same criteria as for enriched pathways (Table S3). We further correlated these potential key metabolites to the gut microbiota through a network analysis approach and found 16 gut‐bacterial ASVs (Figure 5C). Among these, seven (ASV53, ASV65, ASV73, ASV100, ASV125, ASV155, and ASV191) were principally correlated to upregulated DAMs, and three of these (ASV100, ASV125, and ASV191) were also associated with downregulated DAMs (Figure 5C). We also assessed correlations between these key gut‐bacterial ASVs and DAMs involved in the isoflavonoid biosynthesis pathways (Figure 5D). Seven ASVs (three *Desulfovibrio*, one *Phascolarctobacterium*, one *Faecalibacterium*, and two uncultured bacteria) were negatively correlated to all five DAMs, while one (*Limosilactobacillus*) was positively correlated to them (Spearman's rank correlation, *p* < 0.05). Interestingly, the ASVs that significantly correlated to all five DAMs in isoflavonoid biosynthesis belonged to the phylum Firmicutes.

### Effects of gut microbiota on host gene expression

We compared the transcriptomes of the lung, heart, and liver tissues among the different populations to further investigate the plateau adaptation of the Tibetan chicken. The transcriptomes of all three tissues varied significantly among the In‐TC, Ex‐TC, and QY populations (Adonis test, *p* < 0.05). According to the clustering patterns in the PCoA plots, the transcriptomes of the two plains populations (Ex‐TC and QY) were similar but clearly separated from the In‐TC group (Figure S15). A total of 2211, 1936, and 537 genes in lung tissues were recognized as differential expression genes (DEGs) in comparisons of In‐TC versus Ex‐TC, In‐TC versus QY, and Ex‐TC versus QY, respectively (Table S4). For heart tissues, the number of DEGs was 1336 (In‐TC vs. Ex‐TC), 1084 (In‐TC vs. QY), and 590 (Ex‐TC vs. QY) (Table S4). Similar DEG numbers were identified in liver tissues (1296 for In‐TC vs. Ex‐TC, 1045 for In‐TC vs. QY, and 489 for Ex‐TC vs. QY) (Table S4). For all three tissues, DEG numbers between the native Tibetan chicken and the plains populations were higher than between the two plains populations, and the variation level in the transcriptome of the lung was higher than that in the heart and liver.

**FIGURE 6:**
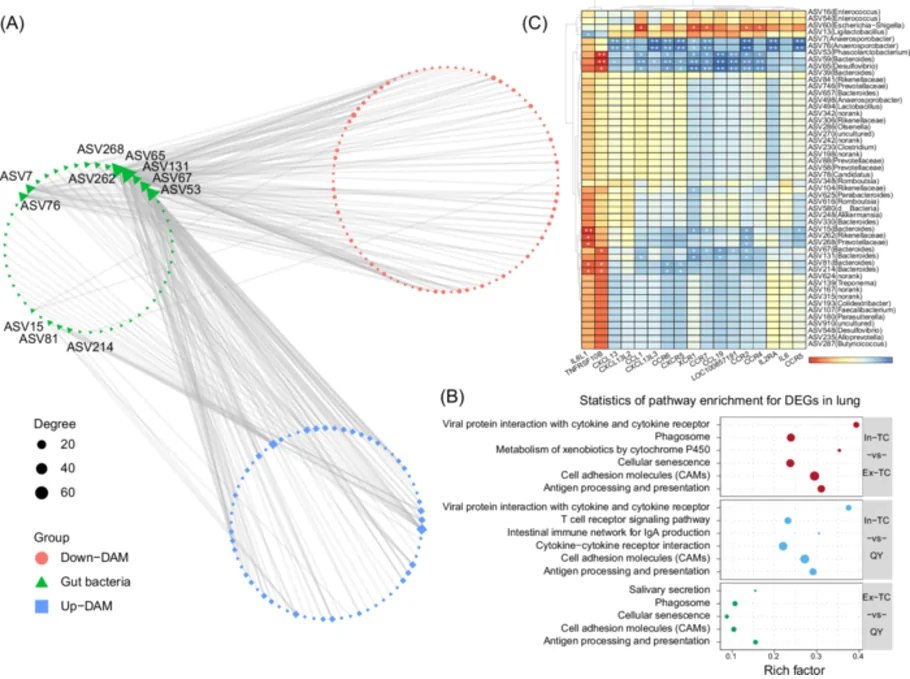
Gut microbiota‐mediated regulation of host gene expression in lung for the plateau adaptation of the Tibetan chicken. (A) Correlation network of gut microbiota and potential key differential expression genes (DEGs) in chicken lung tissues. (B) KEGG enrichment analyses based on DEGs identified from lung tissues of different comparisons. (C) Heatmap showing the correlations between key gut bacterial ASVs and DEGs involved in the viral protein interaction with cytokine and cytokine receptor pathway. “++” and “+” represent *p*‐values <0.01 and <0.05, respectively. ASVs, amplicon sequence variants; KEGG, Kyoto Encyclopedia of Genes and Genomes.

As in the transcriptome analysis, we assumed that DEGs detected in comparisons between the plateau and plains populations but not between the two plains populations could be important for the plateau adaptability of Tibetan chicken. Accordingly, we recognized a total of 410 upregulated and 915 downregulated DEGs as potential key DEGs in lung tissues, together with 172 (up) and 387 (down) in heart tissues and 269 (up) and 386 (down) in liver tissues (Table S5). We further analyzed correlations between the gut microbiota and these potential key genes in the lung by network analysis and found that only a few key DEGs showed significant correlations to gut bacterial ASVs (Figure 6A). For the lung, there were 12 candidates, among which two (ASV7 and ASV76) were specifically correlated to downregulated genes, three (ASV15, ASV81, and ASV214) were specifically correlated to upregulated genes, and the others were correlated to both. We found one Kyoto Encyclopedia of Genes and Genomes (KEGG) pathway, viral protein interaction with cytokine and cytokine receptor, to be potentially important because of its enrichment in the In‐TC group compared to the Ex‐TC and QY populations (Figure 6B). Most DEGs involved in this pathway were significantly downregulated in In‐TC lung tissue compared to those in Ex‐TC and QY samples (Figure S16). Interestingly, the expression levels of DEGs in this pathway were significantly correlated to the relative abundances of the 12 potential key gut bacterial ASVs noted above (Figure 6C). These results indicate the importance of a gut–lung axis in the plateau adaptation of Tibetan chicken.

**FIGURE 7:**
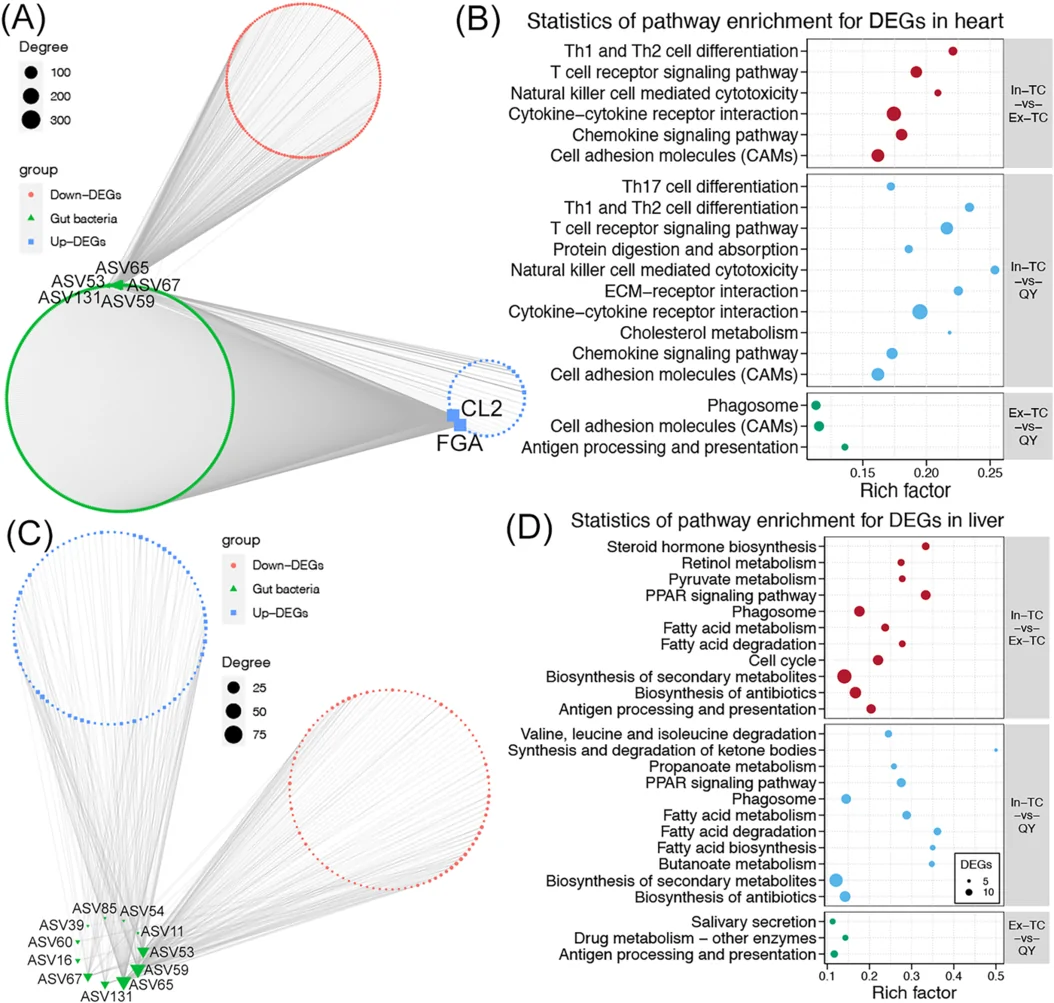
Gut microbiota‐mediated regulation of host gene expression in heart and liver for the plateau adaptation of the Tibetan chicken. (A) Correlation network of gut microbiota and potential key differential expression genes (DEGs) in chicken heart tissues. (B) Kyoto Encyclopedia of Genes and Genomes (KEGG) enrichment analyses based on DEGs identified from heart tissues of different comparisons. (C) Correlation network of gut microbiota and potential key DEGs in the liver. (D) KEGG enrichment analyses based on DEGs identified from liver tissues of different comparisons.

We also performed the same type of network and KEGG enrichment analyses for heart and liver samples. In the heart, a relatively large number of gut bacterial ASVs was significantly correlated to potential key DEGs (Figure 7A). Notably, most of these ASVs were significantly correlated to two upregulated DEGs (FGA and CL2), while another five (ASV53, ASV59, ASV65, ASV67, and ASV131) were correlated to diverse DEGs (Figure 7A). Four KEGG pathways were enriched in heart samples of the In‐TC group compared to the plain populations: Th1 and Th2 cell differentiation, T cell receptor signaling pathway, natural killer cell‐mediated cytotoxicity, and cytokine‐cytokine receptor interaction (Figure 7B). For the liver, 10 gut bacterial ASVs were found to be significantly correlated to potential key DEGs (Figure 7C), and six KEGG pathways [biosynthesis of antibiotics, biosynthesis of secondary metabolites, fatty acid degradation, fatty acid metabolism, phagosome, and peroxisome proliferator‐activated receptors signaling] were enriched in the In‐TC group compared to the plains populations (Figure 7D). It is noteworthy that we identified five gut bacterial ASVs (ASV53, ASV59, ASV65, ASV67, and ASV131) as potential key ASVs in all three investigated axes (gut–lung, gut–heart, and gut–liver) of the Tibetan chicken.

## 3 DISCUSSION

We found significant variations in the diversity, composition, and assembly mechanisms of gut microbiota among the different chicken populations. Interpopulation variations in gut microbiota have been reported in several animals, including humans, pigs, and poultry [28, 29, 30, 31], and increased species turnover over time was found in the gut microbiome of diverse human populations [32]. Our results revealed that species turnover is also the dominant mechanism driving population variations in the gut microbiota of chickens. Previous studies have shown that host genetic variation can shape the composition of chicken gut microbiota [33]. The effects of population variations in gut microbiota mainly manifest in constraining colonization by specific microorganisms [34]. This mechanism can partially explain the significant differences in richness among gut microbiota in different chicken populations, as well as the dominant roles of several levels of assembly diversity observed in this study. However, there are many biotic and abiotic factors in addition to host genetic variation that contribute to variations in gut microbiota, such as age, health status, diet, geography, and environmental exposure [35]. The chickens used in our study were all healthy and of the same age, allowing us to exclude some physiological factors that could influence their gut microbiota. More importantly, all five plains populations were raised on the same farm with consistent diet and environmental conditions. The comparatively minor differences in gut microbiota between these populations indicate that living conditions are more important than breed in determining the microbiota assembly of chickens.

The diversity within the gut microbiome plays a crucial role in maintaining community stability. In microbial ecosystems, such as gut microbiomes, stability emerges from complex interactions among diverse taxa [36]. These communities exhibit resilience through mechanisms such as redundancy (where multiple species perform similar functions) and the presence of rare taxa that can buffer against disturbances [37]. Studies have shown that gut microbiomes with high taxonomic diversity and interconnectedness among community members exhibit robust stability when nutrient availability or physical conditions shifted [38]. In addition, the interactions within these microbial communities often follow macroecological patterns, indicating that stability can be maintained even amidst significant changes [39]. In this study, we observed a remarkably lower proportion of shared ASVs in the gut microbiota of In‐TC population compared to other populations that live in the farms on the plains. This phenomenon indicates more diverse taxa in the gut microbiota of In‐TC individuals, which could reflect the advantages of environmental adaptation of this population.

The In‐TC and Ex‐TC populations shared the same genetic background but were raised in different geographic locations. Differences in gut microbiota between these two populations can reveal the effects of habitat factors [40]. Our results indicate that variation in the local species pool was the dominant driver of interpopulation differences. The environmental microbiome is known to act as a source pool for the gut microbiota of animals [41]. The geographical and climatic characteristics of the Qinghai‐Tibet Plateau have created a microbial community that is distinct from that of lower elevations [42], which can result in significant differences in local species pools between plateau and plains habitats. In addition, different diets for In‐TC and Ex‐TC populations can introduce different microbes into the intestinal tract [43]. A relatively narrow phylogenetic distribution of gut microbes was observed in the In‐TC samples. This may also be attributable to the differences in local species pools between the plateau and plains habitats, as specific microbes adapted to low temperature and low oxygen level could be selectively enriched in plateau habitats.

Knowledge of assembly mechanisms of gut microbiota in animals is important for improving our understanding of the relationships between gut microbiota and host health [44]. From a meta‐community perspective, the biotic and abiotic factors influencing the community assembly can be classified into deterministic and stochastic processes [45]. Our findings demonstrate that the gut microbiota of Tibetan chickens living in the plateau environment is dominated by stochastic processes. Such an assembly may represent a more balanced state that enhances the microbiota's resistance to external stresses [46]. In addition, we found a more complex gut microbial network in the In‐TC population compared to the Ex‐TC individuals. High microbiota network complexity often indicates greater robustness against external perturbations [47]; an example is the changes in gut microbiota of healthy pigs with increasing age [48]. We also found higher functional redundancy of gut microbiota in the In‐TC population compared to the Ex‐TC samples, which is indicative of a greater ability to resist strong external stress [49].

One of the objectives of this study was to explore the characteristics of environmental adaptation in Tibetan chickens. Two important genes, *CL2* and *FGA*, were found in the heart tissues of Tibetan chickens and were significantly downregulated in the In‐TC samples compared to those in the plain populations. The *FGA* gene encodes the α component of fibrinogen, which in turn regulates the formation of blood clots [50]. *CL2* encodes the protein collectin‐11, which is involved in the complement pathway and forms oligomers and deposits [51, 52]. This suggests that Tibetan chickens living in high altitudes may have a reduced formation of polymers in the blood, lower blood viscosity, and increased blood oxygen transfer efficiency, increasing their adaptation to hypoxic plateau conditions. In addition, we detected a significant enrichment of the fatty acid degradation pathway in liver tissues of the In‐TC group, with a significant upregulation of all DEGs (Figures S17–18). The liver is the central organ in fatty acid metabolism, in which fatty acids are oxidized to produce adenosine triphosphate [53]. A strong fatty acid degradation ability in the liver of Tibetan chicken may indicate more effective in keeping energy metabolism in liver tissues, supporting survival in harsh conditions.

It is generally assumed that Tibetan chickens surviving in harsh environments have unique evolutionary advantages that enable them to outcompete other populations. If this is correct, however, why does the breed display long‐term adaptations to domestication when raised at lower altitudes? Some findings of our study may shed light on the evolutionary perspective of this. We found that several isoflavonoids were more abundant in the intestinal tract of plains chickens than in In‐TC individuals. These isoflavonoids are related to resistance to pathogen invasion and elimination of excessive oxygen stress [54, 55]. More importantly, our transcriptome results of heart, liver, and lung tissues all showed that multiple adaptive immunity‐related pathways (related to cytokines and T‐cell activation) were enriched and that most of the DEGs among them were significantly upregulated in the plains populations compared to the In‐TC individuals (Figures S16, S19–23). High cytokine and T cell activity are beneficial for pathogen resistance and maintenance of host health [56]. Our results indicate that the plains populations had more effective immune systems than the plateau population. The impacts of urbanization and human activities on the vast and sparsely populated Qinghai‐Tibet Plateau are much less severe than on other more populous locations such as coastal cities. Thus, chickens living on urban farms face more frequent and severe health threats, and an enhanced activation of the immune system may be a response to these influences. This strategy is determined by environmental adaptability rather than innate genetic characteristics, just as humans who have been vaccinated can more effectively resist epidemic diseases [57].

Finally, we detected five core gut microbes that were significantly correlated to the immune activities involved in the gut‐organ axis in all populations, all belonging to the phylum *Firmicutes*. In a healthy state, the host's immune response to intestinal microbiota is orchestrated to maintain key features of host‐microbe symbiosis [58, 59]. *Firmicutes* in gut microbiota have been reported to be related to the host's immune system. For example, colonization by Firmicutes was found to mediate the gut immune maturation in human and mouse [60]. Another study revealed that *Bacteroidetes* and *Firmicutes* have distinct effects on intestinal immunity by differentially inducing primary and secondary responses [61]. However, the relationship between Firmicutes and the host's immune system is complex, and the specific mechanisms underlying this interaction are not clear. Host adaptive immunity has also been observed to alter the gut microbiota by causing an enrichment of *Firmicutes* [62]. Some classifications in the intestinal microbiota are generally considered beneficial, called probiotics, which are defined as active microorganisms (*Lactobacillus*, *Bifidobacterium*), that have beneficial effects on the host's health [63]. Probiotics from intestinal microbiota can enhance the host's immune function, secrete compounds that aid digestion, prevent the colonization of pathogens, and regulate gastrointestinal function [64]. Thus, to fully understand the role of *Firmicutes* in host adaptation, further studies are required to explore the molecular mechanisms behind this interaction by isolating and developing *Firmicutes* as effective food additives in response to high‐altitude adaptation. The core gut microbes identified in our study may also provide candidate resources for screening probiotics to improve the resistance of chickens to extreme environments.

While our multi‐omics approach provides comprehensive insights into the gut microbiota–host interactions across altitudinal populations, we acknowledge that the modest sample size (*n* = 10 per group) represents a key limitation. Small cohorts may reduce statistical power to detect subtle but biologically relevant differences, and future studies with larger cohorts are needed to validate these preliminary results. Additionally, our study is limited to the effects of high‐altitude adaptation on the relationship between gut microbiota and chicken samples, reproduction, and environments. Functional validation of identified pathways and specific microbiota isolation would strengthen our understanding of high‐altitude adaptation‐related molecular changes to develop functional additives.

## 4 CONCLUSION

We carried out a multi‐omics study to explore the environmental adaptation mechanisms of chickens, with a special focus on the Tibetan breed. We found significant effects of population origin and habitat on gut microbiota, in which the local species pool and species turnover acted as the dominant drivers of variation. When compared to populations living in a lower‐altitude environment, Tibetan chickens living on the plateau were found to have a gut microbiota with a greater capacity to resist external stresses. Our study also elucidated the environmental adaptation strategies via the gut‐organ axis, as exemplified by more effective energy absorption in plateau chickens and stronger immune activities in plains chickens. Our findings contribute valuable insights into the adaptation of chicken gut microbiota to high‐altitude environments and provide candidate resources for the future exploitation of probiotic products to improve resistance to hypoxic environments.

## 5 METHODS

### Study animals, sample collection

To minimize the influence of diet and breeding environment, the cohorts of all five lowland chicken populations were raised in the same low‐altitude location (Yangzhou, Jiangsu, <20 m) with consistent feeding environment, management, and nutritional conditions. While the In‐TC population was raised on the plateau (Lhasa, Tibet, 3700 m) (Table S6). The chickens raised in high‐ and low‐altitude locations were fed the same diets ad libitum based on the composition of corn and soybean meal throughout the experimental period. Chicken diet was mainly provided with 2900 kcal/kg of metabolizable energy (ME) and 19%–22% of crude protein (CP) in brooding period, 2750 kcal/kg ME and 17%–20% CP in rearing period, and 2650 kcal/kg ME and 14%–16% CP in laying period. All animals were 300 days old, healthy, and had not received any medication for at least 3 months before sampling. We picked 10 individuals from each population and collected their cecum contents and heart, liver, and lung tissues under sterile conditions. The sampling locations for six populations were consistent: the left side of the liver and lung, and the apex of the heart were taken. Samples were added separately to 1.5 mL sterile polypropylene tubes and immediately snap‐frozen in liquid nitrogen until further analysis.

### Blood physiological indices measurement

The RBC indices, including RBC count, HCT, HGB, MCH, MCHC, and MCV, were measured via the CBC test by the Sysmex cell counter (Abbott, model Alcyon 300).

### DNA extraction and gut microbiota sequencing

We extracted the microbial DNA of each cecum content sample using a DNA Stool MiniKit (QIAGEN, Venlo) according to manufacturer's instructions, performed agarose gel electrophoresis (1.5%) to validate the extraction, and used a NanoPhotometer Classic instrument (Implen, Munich, Germany) to measure the purity and concentration of the DNA. We used primers 27F and 1492R to amplify full‐length bacterial 16S rDNA. Polymerase chain reaction amplification, amplicon purification, and library construction followed Gao et al. [65]. Sequencing was carried out on a PacBio Sequel platform by Shanghai Biozeron Biotechnology Co., Ltd. PacBio raw reads were processed to obtain demultiplexed circular consensus sequence (CCS) reads using SMRT Link Analysis software (v. 9.0). After assigning the CCS reads to specific samples according to their unique barcode, data processing (quality control, pair‐end read assembly, clustering, and counting) was done using QIIME2 based on the ASV strategy [66]. ASVs with only one read number (singletons) were removed, and the remaining ASVs were taxonomically annotated according to the SILVA database (Release 138) [67]. Finally, we normalized the ASV abundance table based on the lowest read number among all samples.

### Nontargeted metabolomics

Based on the results of the gut microbiota analyses, we selected three populations (In‐TC, Ex‐TC, and QY) to perform nontargeted metabolomics. To extract the metabolites, we resuspended 100 mg of chicken cecum contents of each individual in 500 μL pre‐chilled 80% methanol with 0.1% formic acid and vortexed the mixture. We then incubated the samples at 4°C for 5 min., and centrifuged them at 15,000 × *g* for 20 min. The supernatant of each sample was diluted to a final concentration of 53% methanol by HPLC grade water, and then centrifuged again at 15,000 × *g* for 20 min. To separate metabolites, supernatants were collected and injected into a Vanquish ultrahigh‐performance liquid chromatography (UHPLC) system (Thermo Fisher Scientific, Waltham, MA, USA) with a Hypesil Gold column (100 mm × 2.1 mm; 1.9 μm). We applied an Orbitrap Q Exactive TMHF‐X mass spectrometer (Thermo Fisher Scientific) to detect metabolites eluting from the UPLC column. The detailed process of UHPLC‐MS/MS followed a previous study [68]. To process peak information from the raw data to quantify metabolites, we employed a Compound Discoverer v. 3.1 (Thermo Fisher Scientific). To obtain accurate qualitative and quantitative results of each metabolite, peaks were matched with the mzVault and MassList databases. We carried out three comparisons of metabolites: In‐TC versus Ex‐TC, In‐TC versus QY, and Ex‐TC versus QY. Student's *t*‐test (*p*‐value < 0.05), VIP value (>1) and fold change (FC, >2 or <0.5) were considered to identify DAMs among different populations. DAMs were annotated using the HMDB and KEGG databases based on searches for accurate DAM *m/z* values.

### Transcriptome sequencing

We extracted total RNA from lung, heart, and liver tissues of samples from the same 3 populations used in nontargeted metabolomics (In‐TC, Ex‐TC, and QY) using the trizol method for RNA‐Seq [69]. Transcriptome libraries (paired‐end 150 bp) were constructed using the TruSeqTM RNA sample preparation Kit (Illumina) and sequenced on an Illumina NovaSeq. 6000 platform at Shanghai BIOZERON Co., Ltd. Quality control for raw reads was done with Trimmomatic (v0.39) using sliding window 4:15 and minlen 75 [70]. Reference genome (https://www.ncbi.nlm.nih.gov/genome/?term=Gallus+gallus) mapping and gene expression calculation were performed with HISTA2 (v2.2.1) [71] and HTSeq (v2.0.3) [72], respectively. As in the nontargeted metabolomics analyses, we carried out three comparisons (n‐TC vs. Ex‐TC, In‐TC vs. QY, and Ex‐TC vs. QY) to identify DEGs. Based on the results obtained using the edgeR R package (v4.4.1) [73], we recognized genes with a log‐trans fold change > 2 and a *p*‐value < 0.05 (adjusted by the false discovery rate) in each comparison as DEGs. Finally, we obtained the KEGG annotation of the DEGs from the reference genome and enrichment analysis using the KOBAS tool (v3.0) [74].

### Statistical analysis

We assessed the diversity of chicken cecal microbiota by four α diversity indices: Chao1, Shannon, Pielou_J, and Pd_faith. Differences in α diversity indices, bacterial abundances, and functional terms of gut microbiota among different populations were tested using Kruskal–Wallis test with the Dunn's test. Before the different tests, the relative abundance of gut bacteria was scaled by the centered log‐ratio transformation. We calculated two types of distance (weighted and unweighted Unifrac) between pairs of microbiota. The unweighted Unifrac distance only considered the species composition without their relative abundances, while the weighted Unifrac distance considered both [25]. Variations in the microbiota composition among different populations were evaluated by the Adonis test, Tukey's HSD test, and PCoA based on weighted and unweighted Unifrac distances. The relative importance of nestedness and turnover on the variation of gut microbiota composition was determined using the betapart method [75], and their differences among different populations were also tested using Tukey's HSD test. We generated Venn diagrams to determine shared ASVs among different populations and displayed their taxonomic composition with a Sankey diagram. The Rao quadratic entropy (a measure of diversity that integrates species‐relative abundances and pairwise interspecies differences) was applied to decompose the gamma diversity of gut microbiota in each population into population alpha diversity and the difference among different populations (beta diversity distance) [76].

Processes governing gut microbiota assembly were quantified by a null model method based on the beta‐nearest taxon index (betaNTI) and the Raup–Crick metric (RC) [77]. Based on the betaNTI, community assembly can be divided into deterministic (|betaNTI| > 2) and stochastic (|betaNTI ≤ 2) processes. Subsequently, deterministic processes can be divided into two categories: homogeneous selection (betaNTI < −2) and heterogeneous selection (betaNTI > 2). Stochastic processes can be assigned to three events: homogeneous dispersal (RC < −0.95), dispersal limitation (RC > 0.95), and drift (|RC| ≤ 0.95) [78].

To further investigate differences in gut microbiota between In‐TC and Ex‐TC populations, we constructed co‐occurrence networks based on co‐relations among gut ASVs. Only ASVs detected in at least 60% of the samples in each population remained for correlation analysis. If the Spearman correlation coefficient was >0.8 and the Benjamini–Hochberg adjusted *p*‐value was <0.01, we considered a correlation between two ASVs statistically robust. We visualized the obtained networks using the Gephi interactive platform and extracted their topological parameters [79]. To assess the stability of microbiota communities, we calculated robustness, vulnerability, and cohesion for co‐occurrence networks of In‐TC and Ex‐TC samples, following a previous study [80]. A higher value of robustness and cohesion but lower values of vulnerability indicate that the co‐occurrence network is more stable. Differences in these stability indices between the In‐TC and Ex‐TC groups were analyzed using Student's *t*‐test. The FRI of individuals from the In‐TC and Ex‐TC populations was determined using the Tax4Fun2 method [81]. Linear regression analysis (ordinary least‐squares) was performed on the coordinated variations in FRI and betaNTI in In‐TC and Ex‐TC populations.

To evaluate differences in metabolite composition and transcriptomes among different populations, we also carried out PCoA based on metabolite abundances or gene expression levels. We generated volcano plots to filter DAMs based on log2(FC) and log10 (*p*‐value) of metabolites. We conducted enrichment analysis with KEGG annotation, based on the DAMs identified from each of the comparisons, and used Venn diagrams to explore the potential key metabolites involved in the environmental adaptation of the populations. We further created correlation networks to illustrate the relationships between DAMs or DEGs with bacterial ASVs based on Spearman's rank correlation, using the same thresholds as for co‐occurrence networks of gut microbiota. Finally, we created a heatmap based on Spearman's rank correlation to show the correlations between potential key metabolites or genes with key gut bacterial ASVs, as determined from the correlation network.

All statistical analyses were performed in R [82](v4.2.2) with the packages vegan [83](v2.6‐8), multcomp [84](v1.4‐26), GUniFrac [85](v1.8), ape [86](v5.8‐1), betapart [87](v1.6), VennDiagram [88](v1.7.3), networkD3 [89](v0.4), picant [90] (v1.8.2), WGCNA [91](v1.73), Tax4Fun2 [92](v1.1.5), LinkET [93](v0.0.7.4), pheatmap [94](v1.0.12), and ggplot2 [95] (v3.5.1).

## AUTHOR CONTRIBUTIONS

**Tao Zeng**: Writing—review and editing; writing—original draft; project administration; visualization; funding acquisition; investigation; conceptualization. **Tiantian Gu**: Methodology; writing—original draft; investigation. **Yongqing Cao**: Methodology; formal analysis. **Yong Tian**: Methodology. **Jianmei Yin**: Methodology. **Peishi Feng**: Conceptualization; project administration. **Hanxue Sun**: Methodology. **Jindong Ren**: Methodology. **Xueying Ma**: Methodology. **Zelong Zhao**: Formal analysis. **Guohui Li**: Methodology. **Li Chen**: Methodology. **Wenwu Xu**: Formal analysis. **Qian Xue**: Methodology. **Wei Han**: Writing—review and editing; project administration. **Lizhi Lu**: Writing—review and editing; project administration.

## CONFLICT OF INTEREST STATEMENT

The authors declare no conflicts of interest.

## ETHICS STATEMENT

All animal experiments were carried out according to the ethical policies and the procedures approved by the Animal Use Committee of Zhejiang Academy of Agricultural Sciences (24ZALAS28, revised in September 2024).

## Supporting information


**Figure S1.** Ratio of amplicon sequence variants (ASVs) successfully annotated at different taxonomic levels.
**Figure S2.** Rarefaction curves of all six populations.
**Figure S3.** Differences in the Shannon and Pielou_J indices of gut microbiota among different chicken populations.
**Figure S4.** The relative importances of nestedness and turnover on beta‐diversity of chicken gut microbiota.
**Figure S5.** Relative abundances of dominant bacterial phyla in gut microbiota of different chicken populations.
**Figure S6.** Differences in the relative abundances of dominant bacterial phyla in gut microbiota among different chicken populations.
**Figure S7.** Average relative abundances of dominant bacterial genera in gut microbiota of all studied samples.
**Figure S8.** Differences in the relative abundances of dominant bacterial genera in gut microbiota among different chicken populations.
**Figure S9.** Venn diagram for identifying the shared ASVs between the In‐TC and Ex‐TC groups.
**Figure S10.** Differences in the sum abundances of shared ASVs between the In‐TC and Ex‐TC groups.
**Figure S11.** Differences in the relative abundances of bacterial genera in gut microbiota of In‐TC and Ex‐TC groups.
**Figure S12.** Differences in the relative abundances of predicted functions in gut microbiota of In‐TC and Ex‐TC groups.
**Figure S13.** Volcano plots for DAMs identification.
**Figure S14.** DAMs colored in the pathway of isoflavonoid biosynthesis.
**Figure S15.** Principal coordinate analysis (PCoA) for different tissues transcriptome among different chicken comparisons. (a) Lung tissue. (b) Heart tissue. (c) Liver tissue.
**Figure S16.** DEGs in lung colored in the pathway of viral protein interaction with cytokine and cytokine receptor.
**Figure S17.** DEGs in liver colored in the pathway of fatty acid biodegradation.
**Figure S18.** DEGs in liver colored in the pathway of peroxisome proliferator‐activated receptors (PPAR) signaling pathway.
**Figure S19.** DEGs in heart colored in the pathway of cytokine‐cytokine receptor interaction.
**Figure S20.** DEGs in heart colored in the pathway of natural killer cell mediated cytotoxicity.
**Figure S21.** DEGs in heart colored in the pathway of Th1 and Th2 cell differentiation.
**Figure S22.** DEGs in heart colored in the pathway of T cell receptor signaling pathway.
**Figure S23.** DEGs in liver colored in the pathway of phagosome.

**Table S1.** Numbers of taxonomic annotation.
**Table S2.** Topological parameters for Co‐occurrence networks of gut microbiota in the In‐TC and Ex‐TC group, respectively.
**Table S3.** Shared and unique differentially abundant metabolites (DAMs) among different population comparisons.
**Table S4.** Numbers of differential expression genes (DEGs) from different tissues among different population comparisons.
**Table S5.** Shared and unique differential expression genes (DEGs) among different population comparisons.
**Table S6.** Information of chicken breeds used in this study.

## Data Availability

The data that support the findings of this study are available from the corresponding author upon reasonable request. The raw reads of PacBio full‐length 16S rRNA sequencing and transcriptome sequencing for all samples have been deposited in the NCBI Sequence Read Archive (SRA) under the accession number PRJNA975664 (https://www.ncbi.nlm.nih.gov/bioproject/PRJNA975664) and PRJNA950191 (https://www.ncbi.nlm.nih.gov/bioproject/PRJNA950191). Metabolomics data have been deposited into the China National GeneBank Sequence Archive (CNSA) of China National GeneBank DataBase (CNGBdb) under project CNP0004383 (https://db.cngb.org/search/project/CNP0004383/). The data and scripts for analysis are saved in GitHub (https://github.com/TaoZeng4009/ZengiMetaOmics2025.git). Supplementary materials (figures, tables, graphical abstract, slides, videos, Chinese translated version, and update materials) may be found in the online DOI or iMeta Science http://www.imeta.science/imetaomics/.
