# Supplementary material for: Multi‐omics analysis of gut‐organ axes reveals the high‐altitude adaptation in Tibetan chicken
Source: IMetaOmics. 2025 Jun 29;2(3):e70038. doi: 10.1002/imo2.70038 (PMC12806142; doi:10.1002/imo2.70038)
Supplement: Supplementary file 1 — Figure S1. Ratio of amplicon sequence variants (ASVs) successfully annotated at different taxonomic levels. Figure S2. Rarefaction curves of all six populations. Figure S3. Differences in the Shannon and Pielou_J indices of gut microbiota among different chicken populations. Figure S4. The relative importances of nestedness and turnover on beta‐diversity of chicken gut microbiota. Figure S5. Relative abundances of dominant bacterial phyla in gut microbiota of different chicken populations. Figure S6. Differences in the relative abundances of dominant bacterial phyla in gut microbiota among different chicken populations. Figure S7. Average relative abundances of dominant bacterial genera in gut microbiota of all studied samples. Figure S8. Differences in the relative abundances of dominant bacterial genera in gut microbiota among different chicken populations. Figure S9. Venn diagram for identifying the shared ASVs between the In‐TC and Ex‐TC groups. Figure S10. Differences in the sum abundances of shared ASVs between the In‐TC and Ex‐TC groups. Figure S11. Differences in the relative abundances of bacterial genera in gut microbiota of In‐TC and Ex‐TC groups. Figure S12. Differences in the relative abundances of predicted functions in gut microbiota of In‐TC and Ex‐TC groups. Figure S13. Volcano plots for DAMs identification. Figure S14. DAMs colored in the pathway of isoflavonoid biosynthesis. Figure S15. Principal coordinate analysis (PCoA) for different tissues transcriptome among different chicken comparisons. (a) Lung tissue. (b) Heart tissue. (c) Liver tissue. Figure S16. DEGs in lung colored in the pathway of viral protein interaction with cytokine and cytokine receptor. Figure S17. DEGs in liver colored in the pathway of fatty acid biodegradation. Figure S18. DEGs in liver colored in the pathway of peroxisome proliferator‐activated receptors (PPAR) signaling pathway. Figure S19. DEGs in heart colored in the pathway of cytokine‐cytokine receptor interaction. F [file IMO2-2-e70038-s002.docx]

**Supporting information to**

**Multi-omics analysis of gut-organ axes reveals the high-altitude adaptation in Tibetan chicken**

**Running title: Gut-organ axes in high-altitude adaptation**

**Tao Zeng^1,2*#^, Tiantian Gu^1,2#^, Yongqing Cao^1,2^, Yong Tian^1,2^, Jianmei Yin^3,4^, Peishi Feng^5^, Hanxue Sun^1,2^, Jindong Ren^1,2^, Xueying Ma^6^, Zelong Zhao^7^, Guohui Li^3,4^, Li Chen^1,2^, Wenwu Xu^1,2^, Qian Xue^3,4^,** **Wei Han^3,4*^, Lizhi Lu^1,2*^**

^1^State Key Laboratory for Quality and Safety of Agro-Products, Key Laboratory of Livestock and Poultry Resources (Poultry) Evaluation and Utilization, Ministry of Agriculture and Rural Affairs, Institute of Animal Husbandry and Veterinary Science, Zhejiang Academy of Agricultural Sciences, Hangzhou, 310021, China

^2^Zhejiang Key Laboratory of Livestock and Poultry Biotech Breeding, Zhejiang Provincial Engineering Research Center for Poultry Breeding Industry and Green Farming Technology, Hangzhou, 310021, China

^3^National Chickens Genetic Resources, Jiangsu Institute of Poultry Science, Yangzhou, 225125, China

^4^Technology Innovation Co., Ltd., Jiangsu Institute of Poultry Science, Yangzhou, 211412, China

^5^Zhejiang University of Technology, Hangzhou, 310014, China

^6^Institute of Animal Husbandry and Veterinary Medicine, Tibet Academy Agricultural and Animal Husbandry Sciences, Lhasa, 850004, China

^7^Shanghai BIOZERON Biotechnology Co., Ltd., Shanghai, 201800, China

^#^ These authors contributed equally: Tao Zeng, Tiantian Gu.

^*^Corresponding authors. Email: [zengtao4009@126.com](mailto:zengtao4009@126.com) (Tao Zeng); [hanwei830@163.com](mailto:hanwei830@163.com)(Wei Han); [lulizhibox@163.com](mailto:lulizhibox@163.com)(Lizhi Lu)


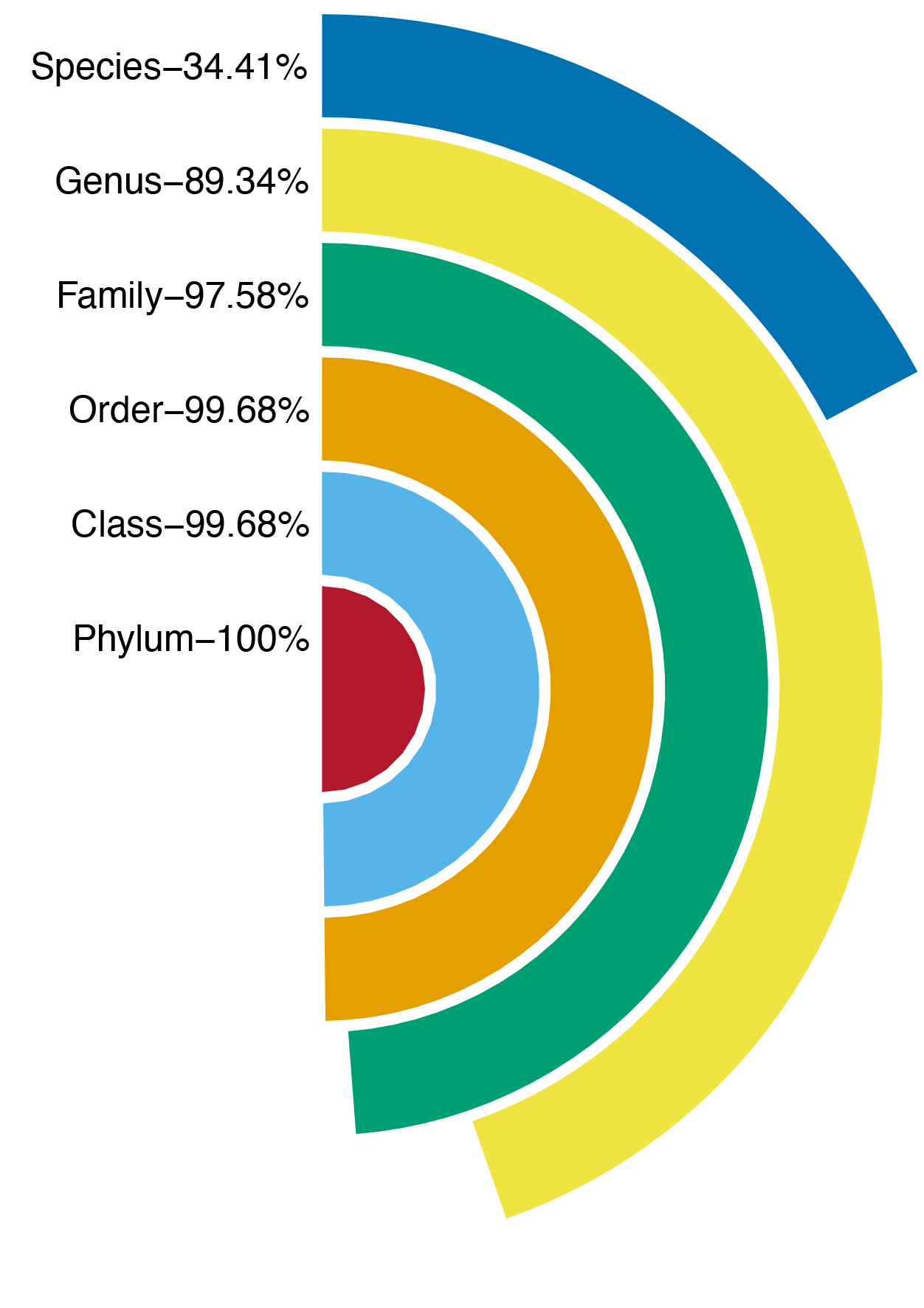


**Figure S1** Ratio of amplicon sequence variants (ASVs) successfully annotated at different taxonomic levels.


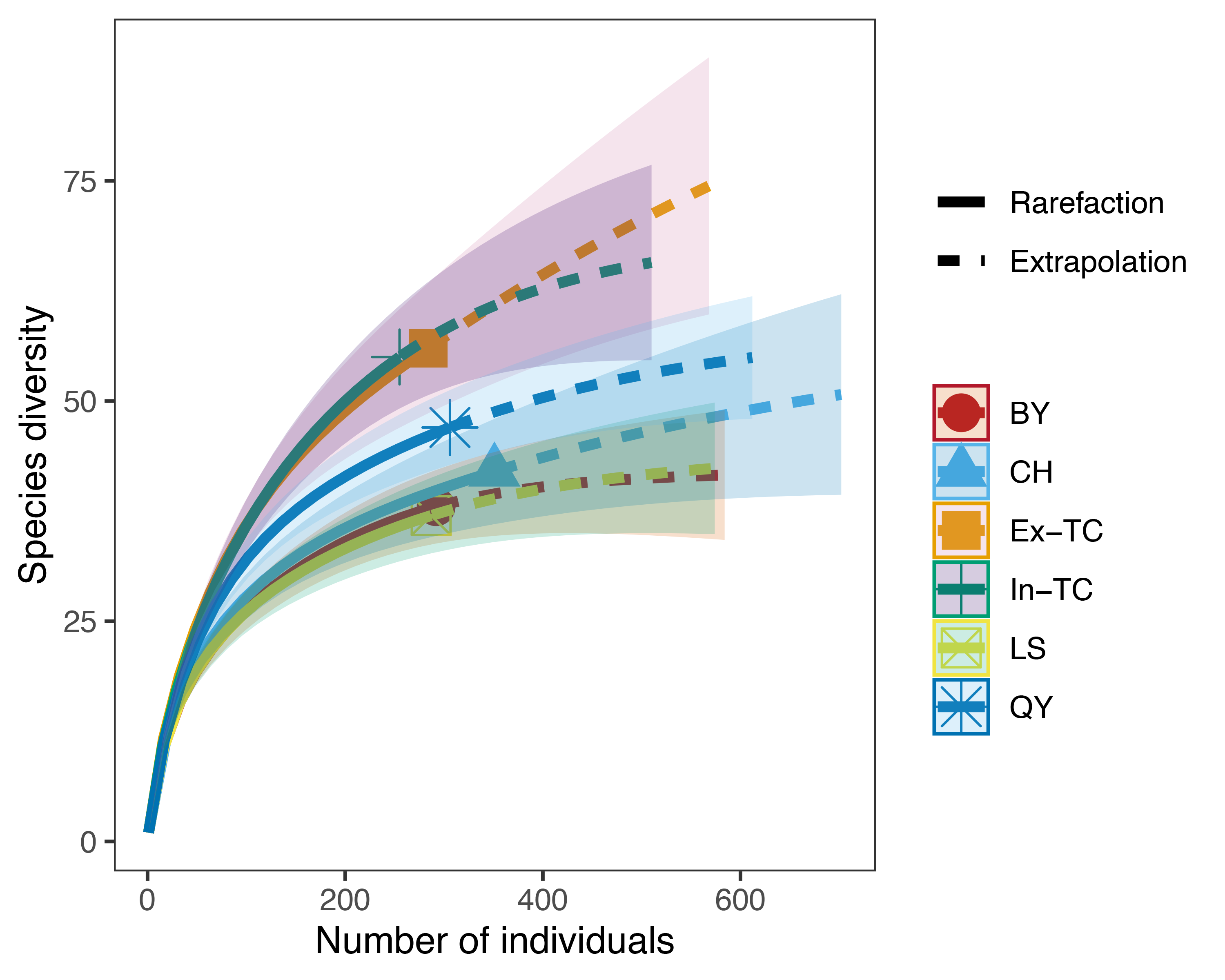


**Figure S2** Rarefaction curves of all six populations. In-TC, Tibetan chicken on the plateau; Ex-TC, Tibetan chicken on the plain; BY, Beijing You chicken; CH, Chahua chicken; LS, Langshan chicken; QY, Qingyuan Ma chicken.


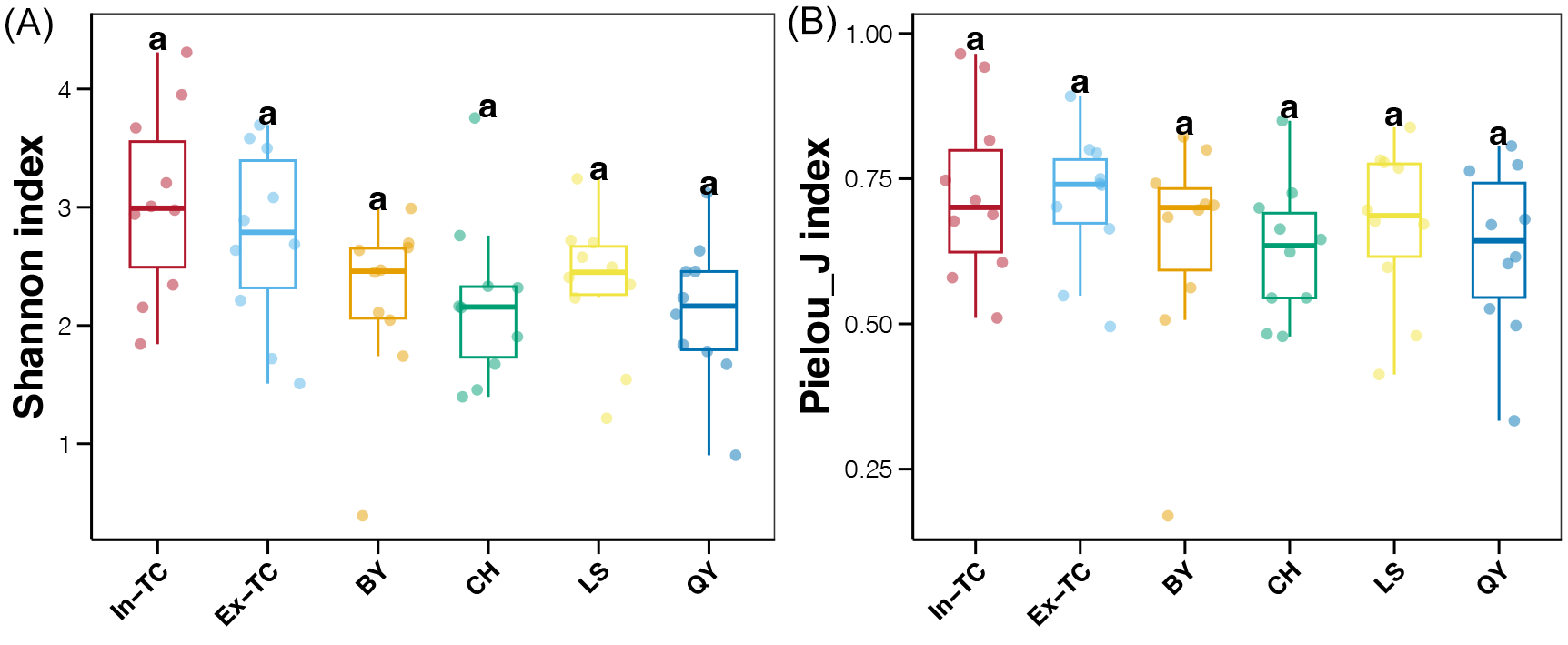


**Figure S3** Differences in the Shannon and Pielou_J indices of gut microbiota among different chicken populations. Different lowercase above the boxes represents the *p*-value of the Tukey's honestly significant difference (HSD) test lower than 0.05 between different groups.


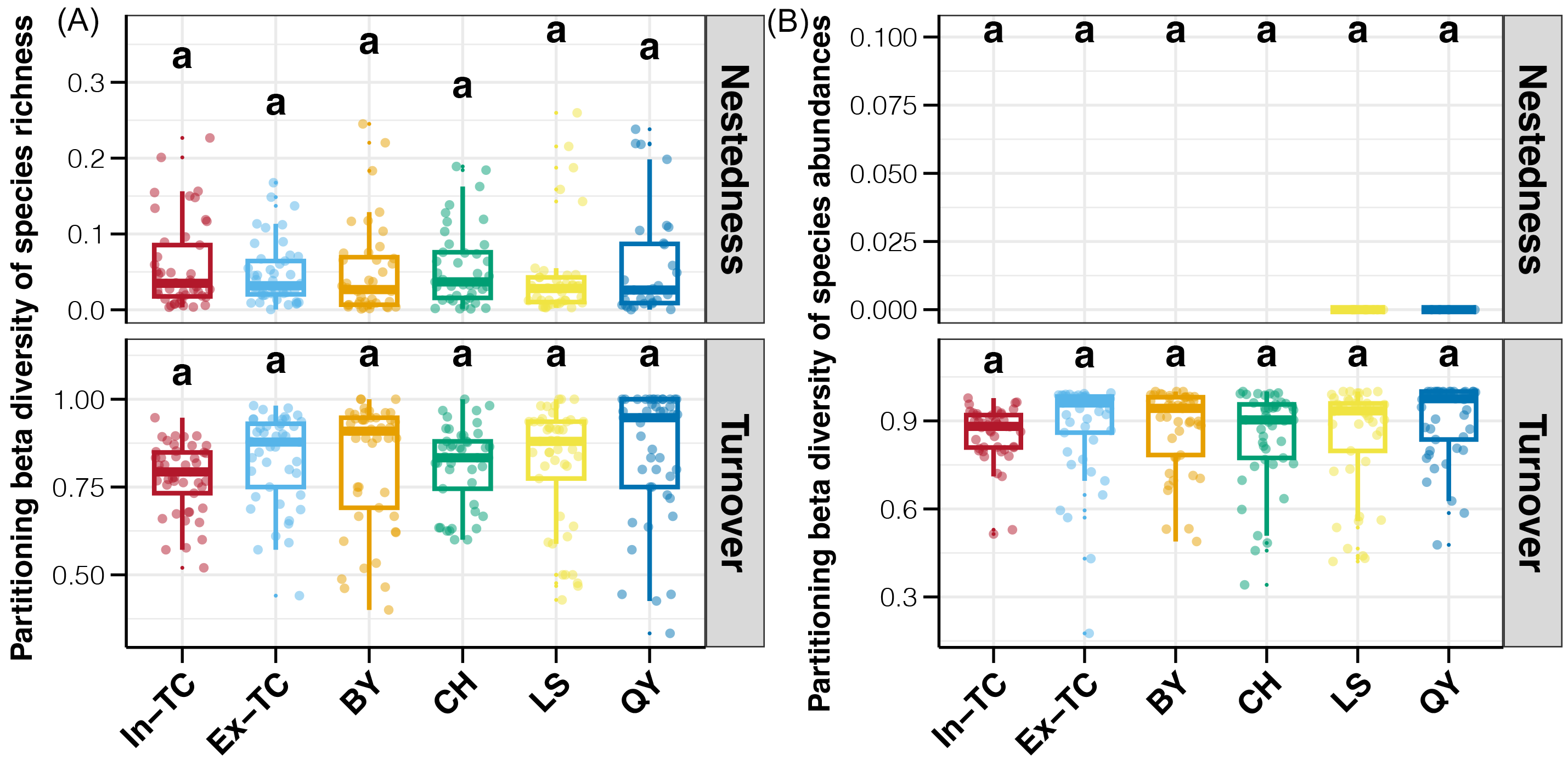


**Figure S4** The relative importances of nestedness and turnover on beta-diversity of chicken gut microbiota. (A) Unweighted unifrac distances. (B) Weighted unifrac distances. Different lowercase above the boxes represents the *p*-value of the Tukey's HSD test lower than 0.05 between different groups.


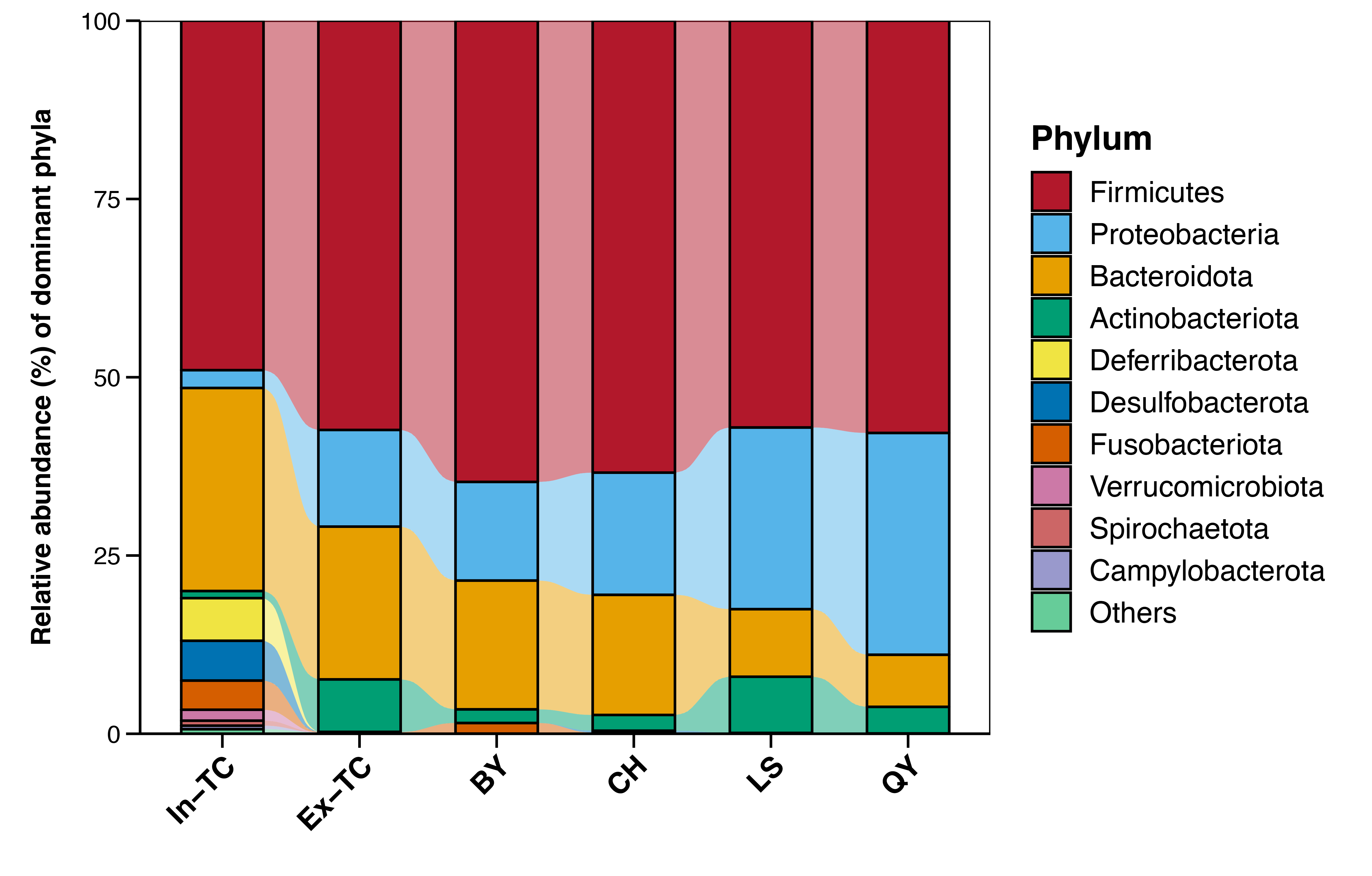


**Figure S5** Relative abundances of dominant bacterial phyla in gut microbiota of different chicken populations.


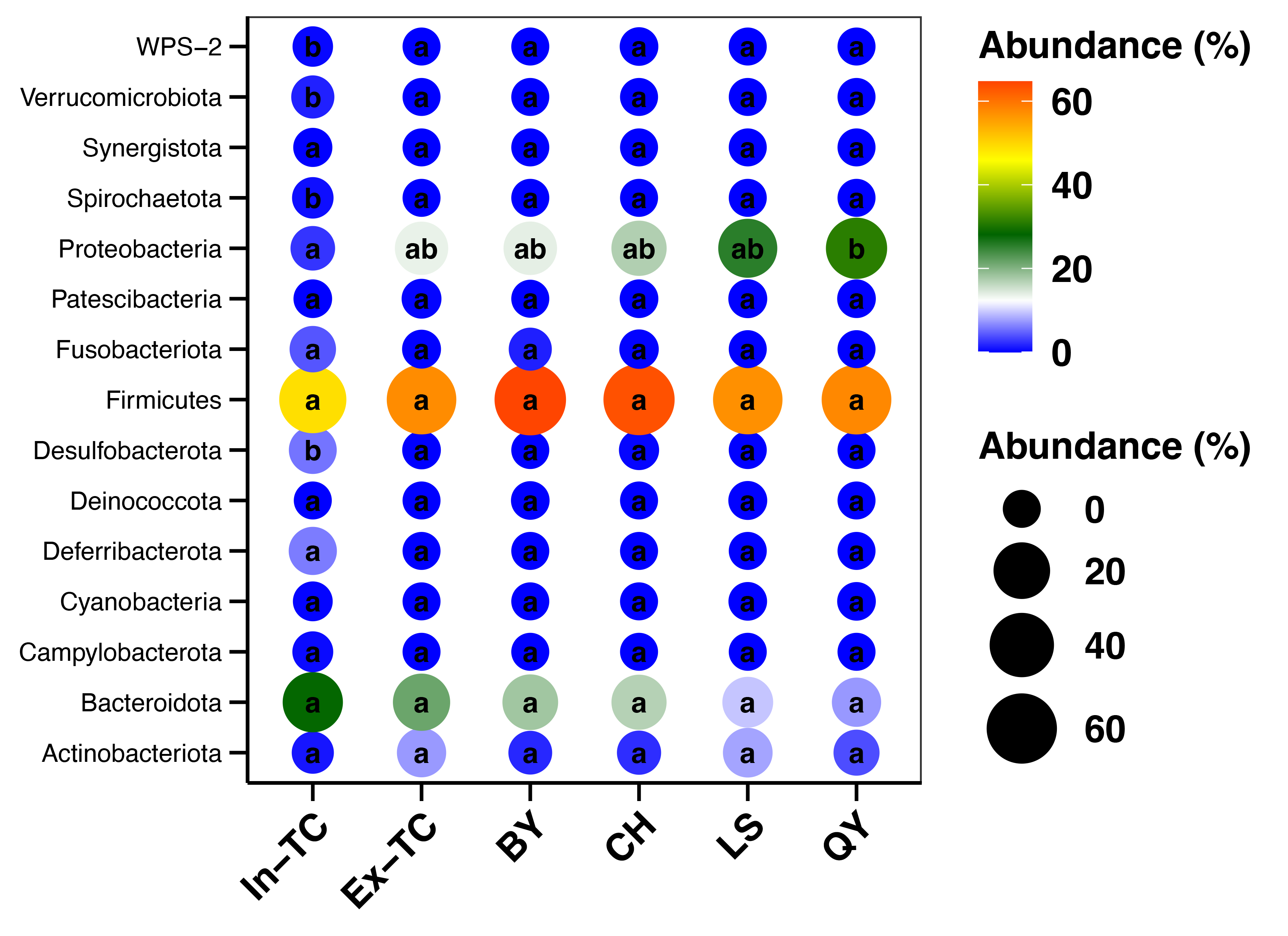


**Figure S6** Differences in the relative abundances of dominant bacterial phyla in gut microbiota among different chicken populations. Different lowercase in points from the same raw represent the *p*-value of the Tukey's HSD test lower than 0.05 between different groups.


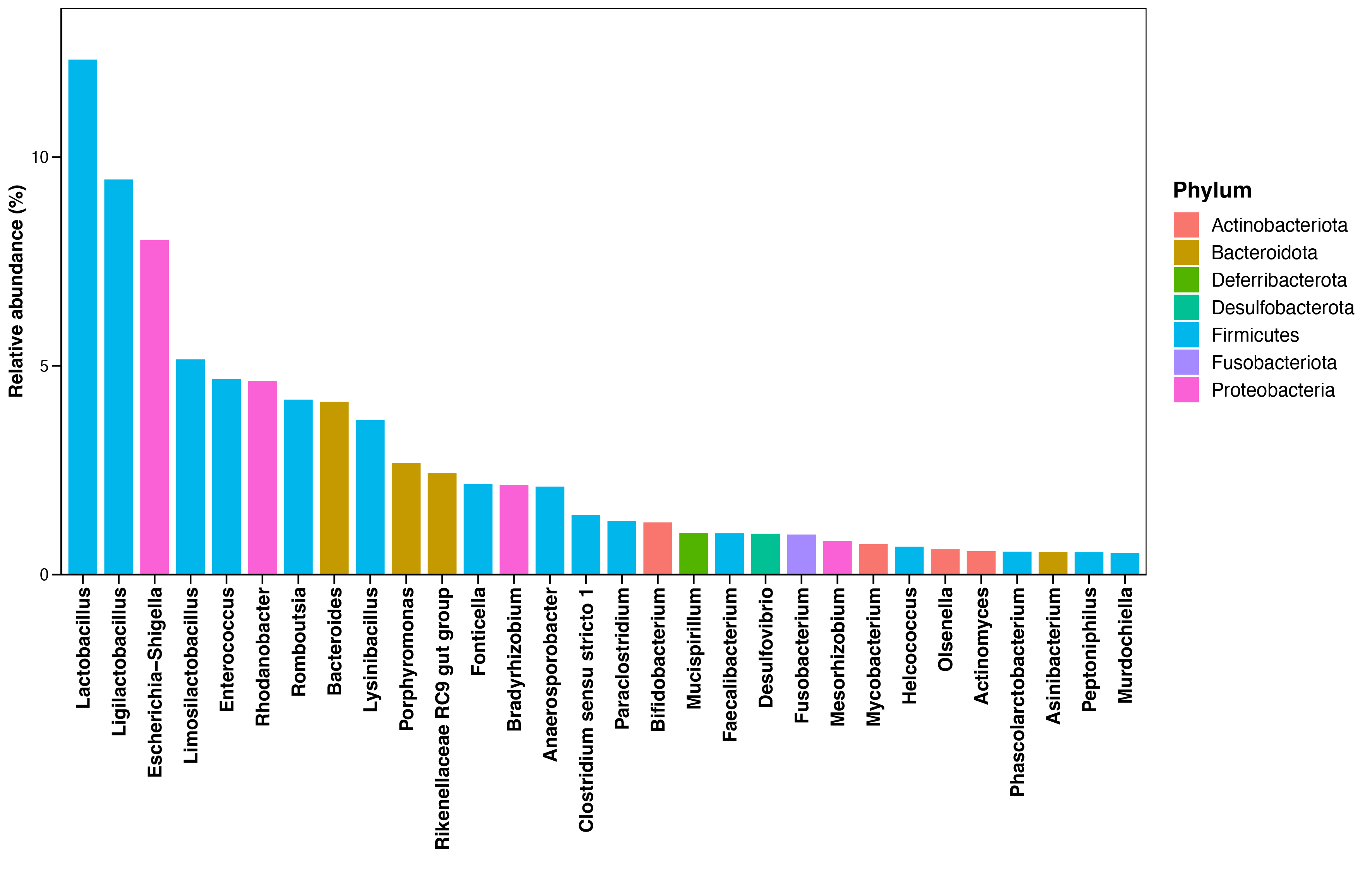


**Figure S7** Average relative abundances of dominant bacterial genera in gut microbiota of all studied samples.


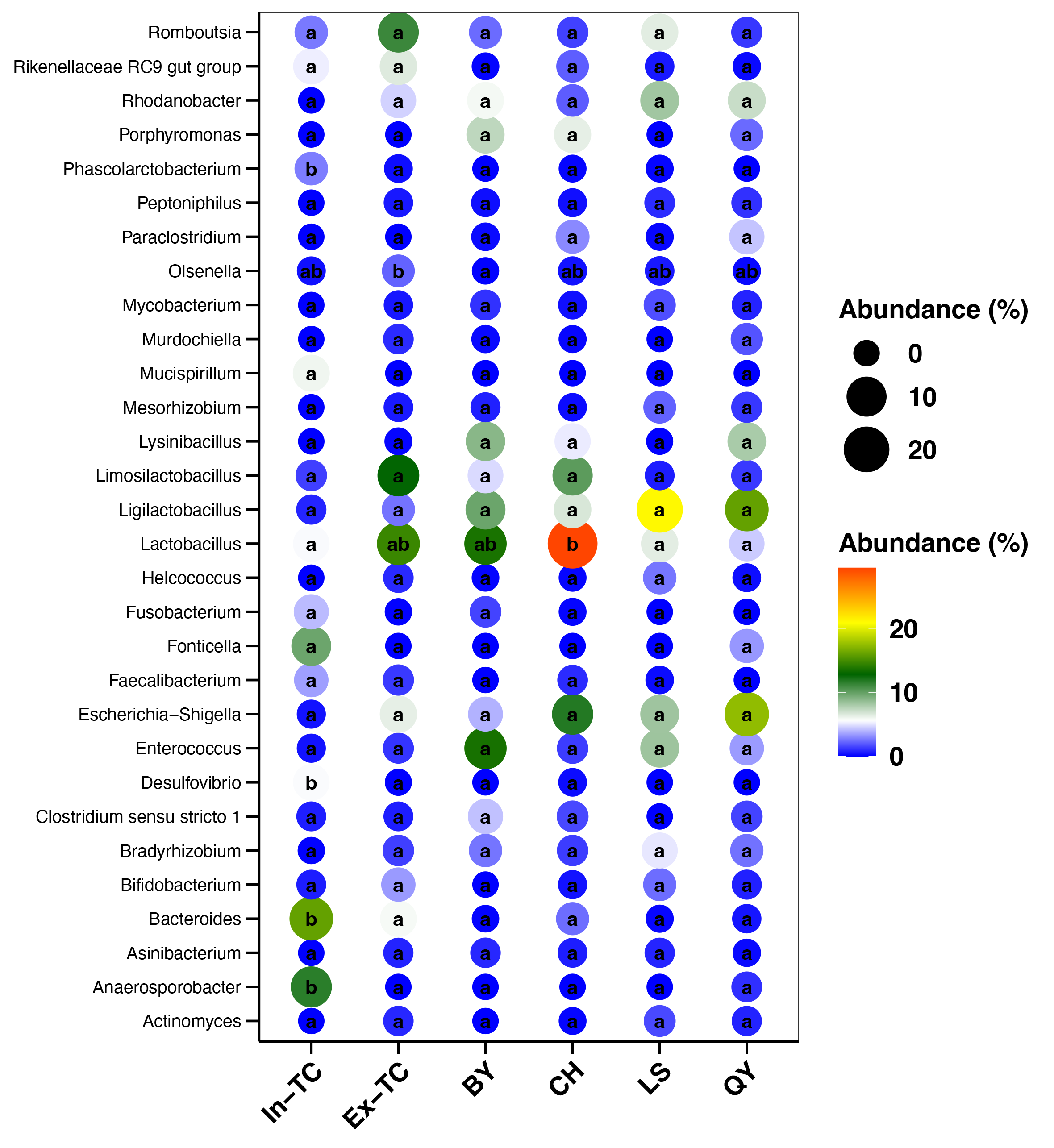


**Figure S8** Differences in the relative abundances of dominant bacterial genera in gut microbiota among different chicken populations. Different lowercase in points from the same raw represent the *p*-value of the Tukey's HSD test lower than 0.05 between different groups.


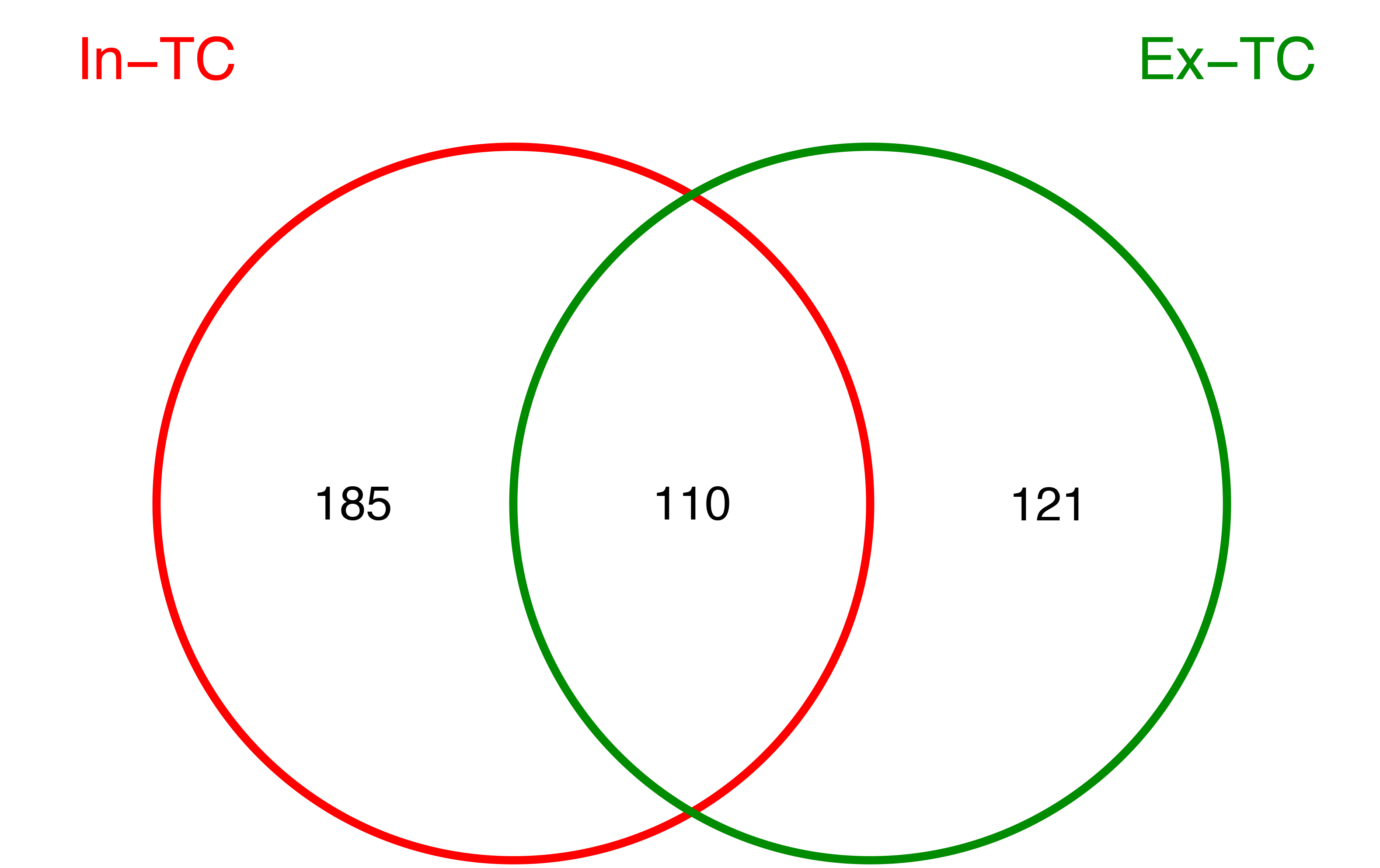


**Figure S9** Venn diagram for identifying the shared ASVs between the In-TC and Ex-TC groups.


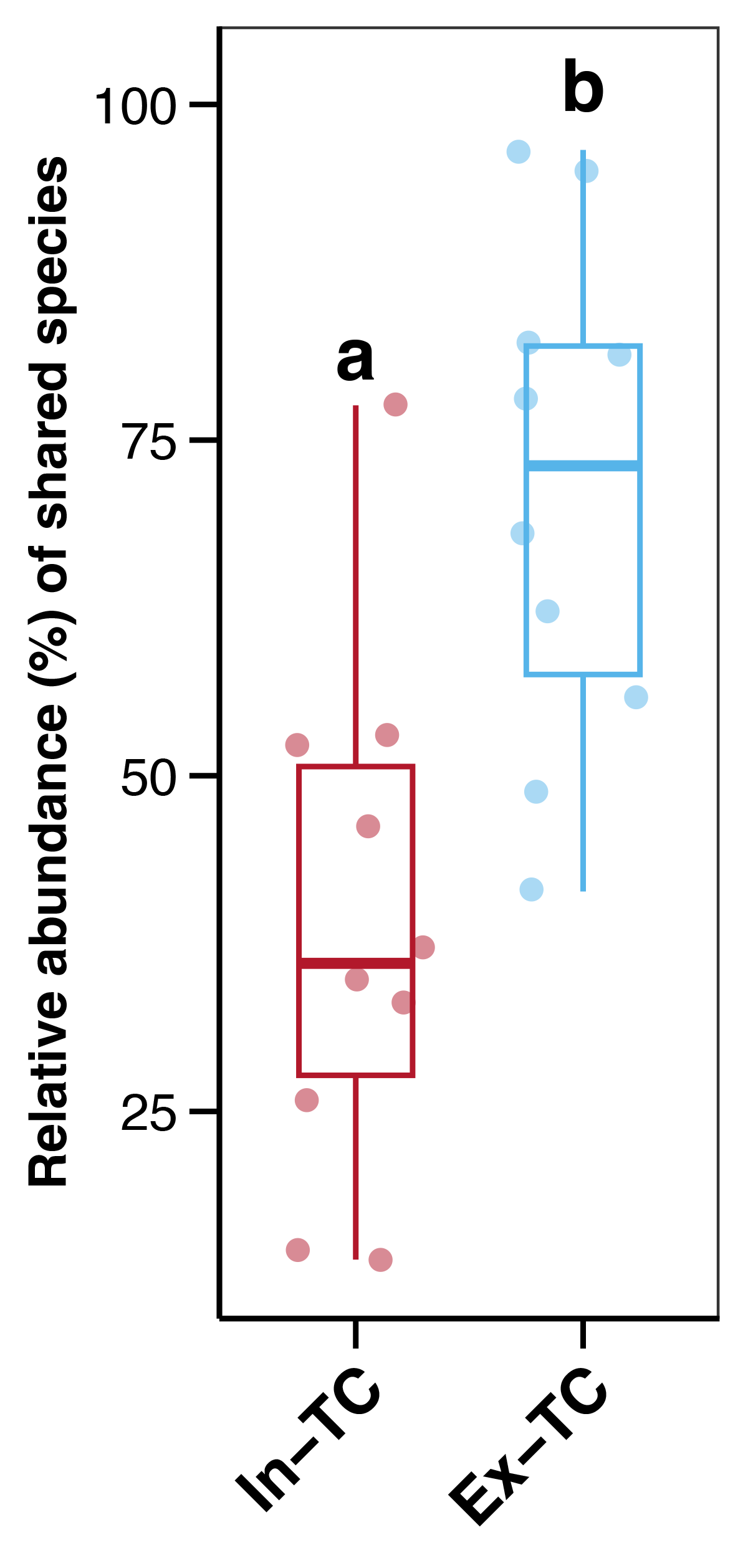


**Figure S10** Differences in the sum abundances of shared ASVs between the In-TC and Ex-TC groups.


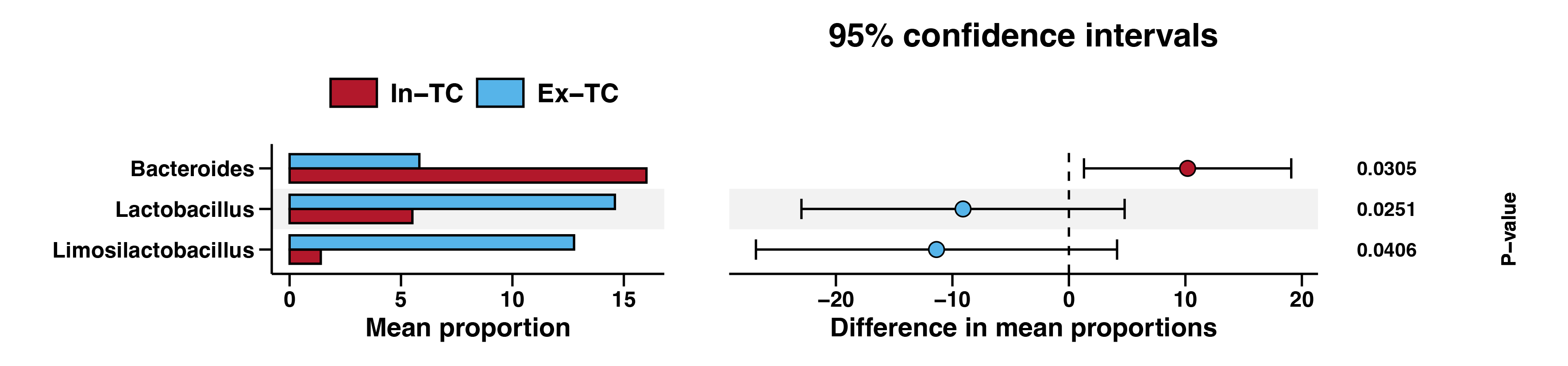


**Figure S11** Differences in the relative abundances of bacterial genera in gut microbiota of In-TC and Ex-TC groups.


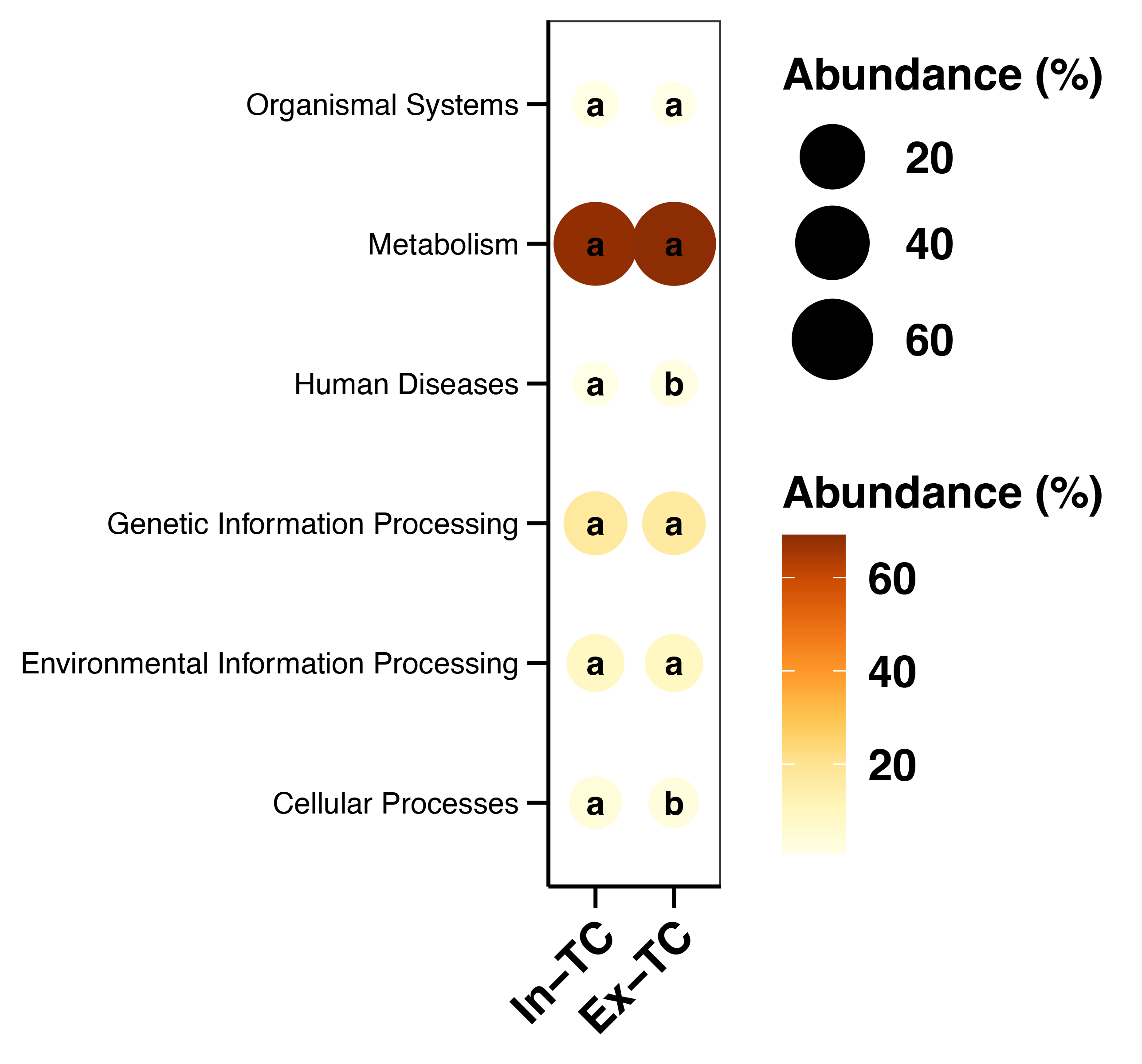


**Figure S12** Differences in the relative abundances of predicted functions in gut microbiota of In-TC and Ex-TC groups. Different lowercase letters inside the points in the same line represent significant differences (*p*-value of Tukey's HSD test < 0.05) between different populations.


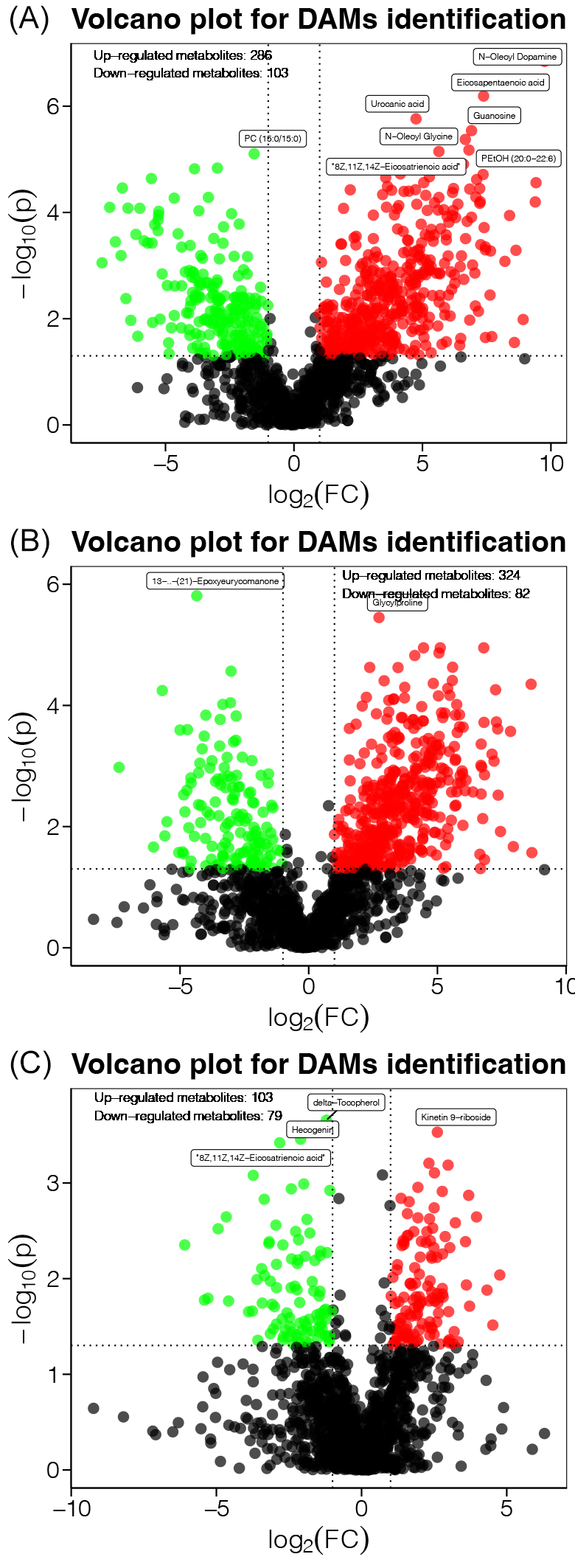


**Figure S13** Volcano plots for DAMs identification. (A) In-TC vs Ex-TC. (B) In-TC vs QY. (C) Ex-TC vs QY.


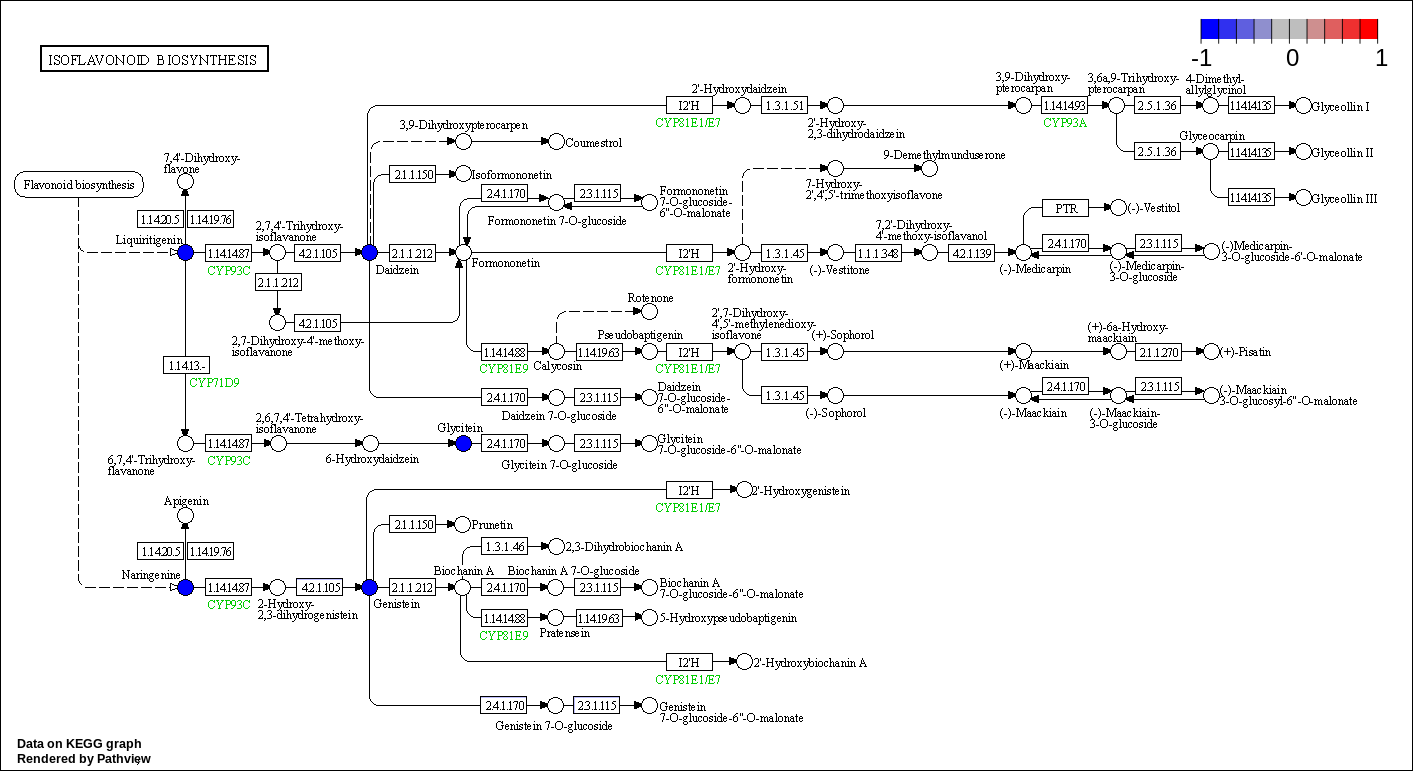


**Figure S14** DAMs colored in the pathway of isoflavonoid biosynthesis. Red represented up-regulated metabolites, blue represented down-regulated metabolites. *p*-value < 0.05.


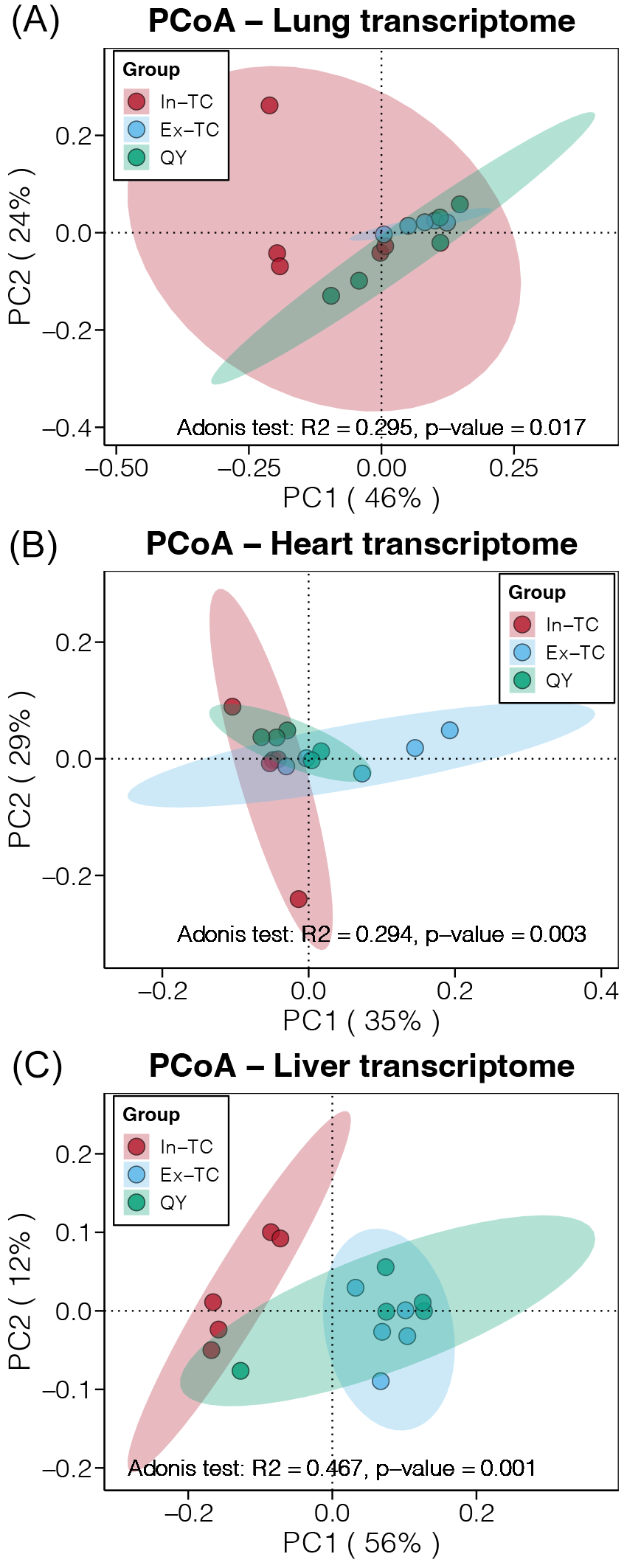


**Figure S15** Principal coordinate analysis (PCoA) for different tissues transcriptome among different chicken comparisons. (A) Lung tissue. (B) Heart tissue. (C) Liver tissue.


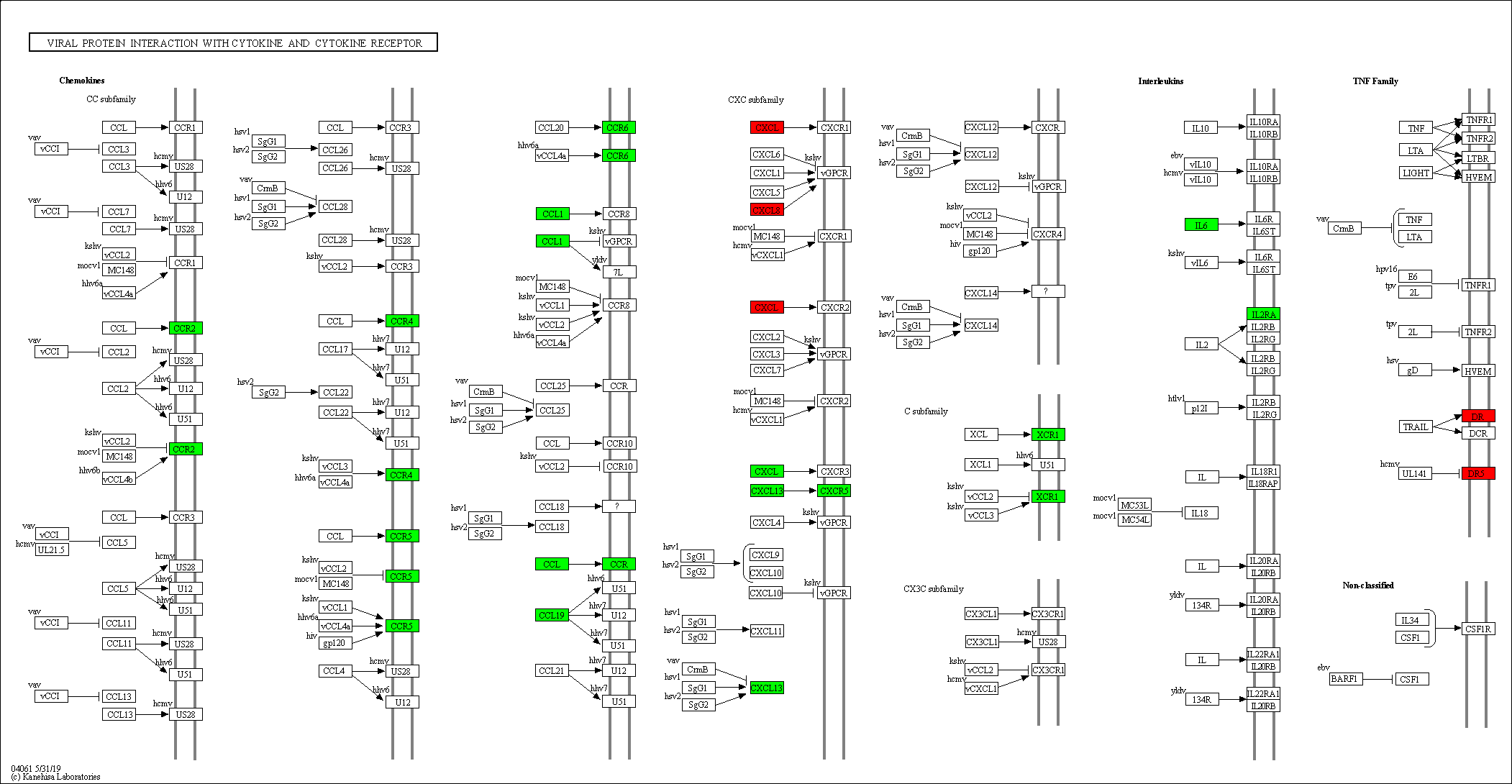


**Figure S16** DEGs in lung colored in the pathway of viral protein interaction with cytokine and cytokine receptor.


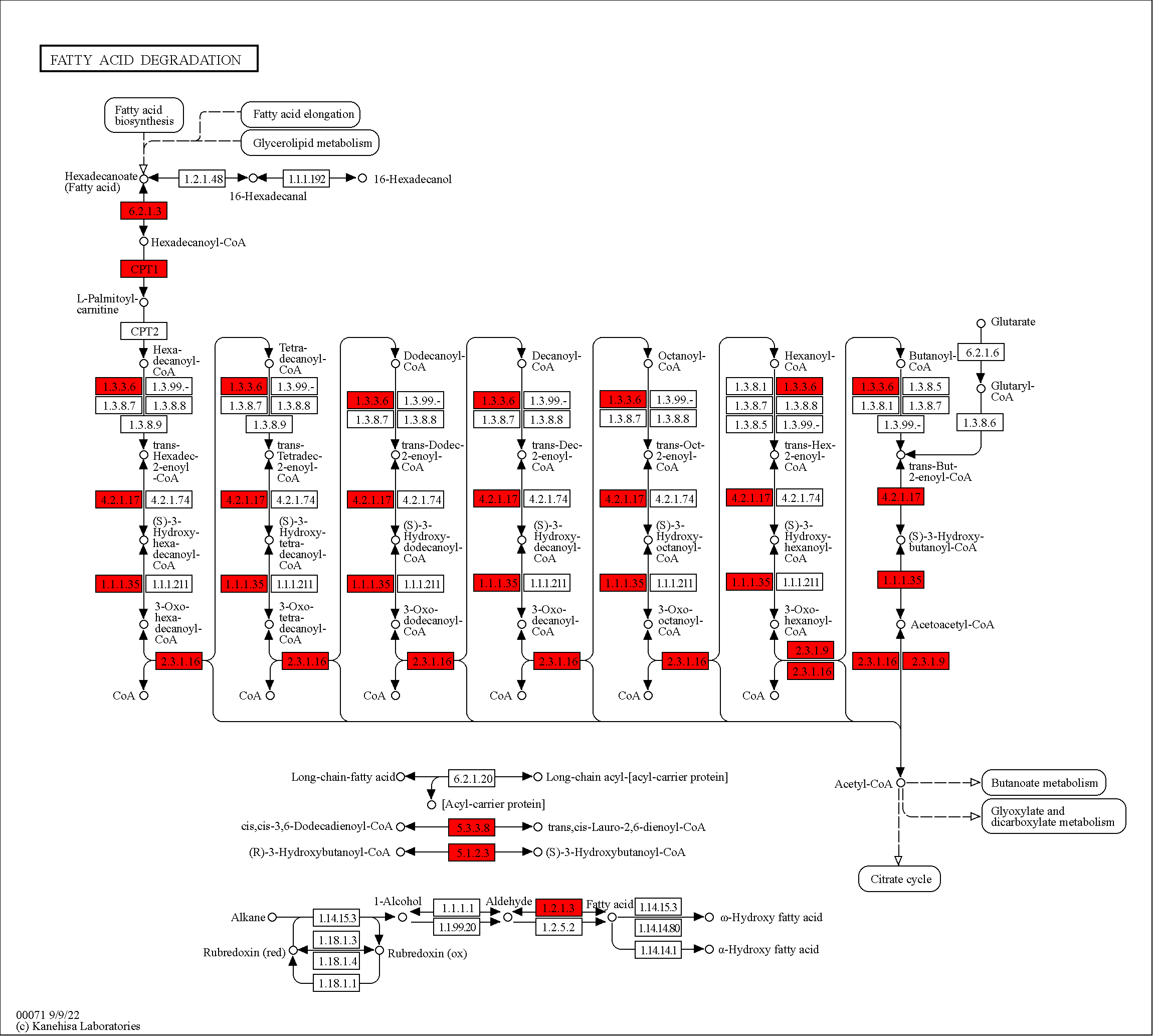


**Figure S17** DEGs in liver colored in the pathway of fatty acid biodegradation.


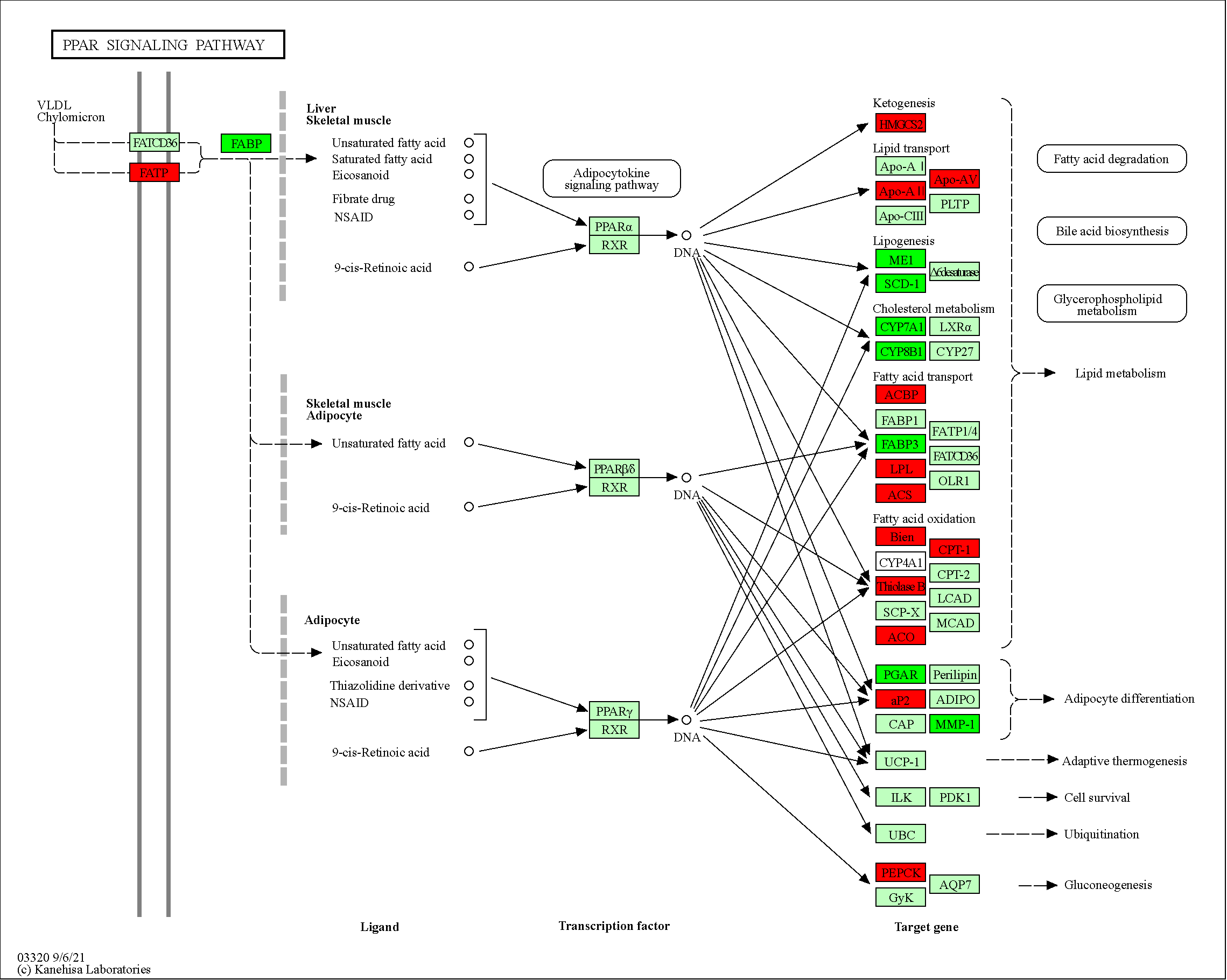


**Figure S18** DEGs in liver colored in the pathway of peroxisome proliferator-activated receptors (PPAR) signaling pathway.


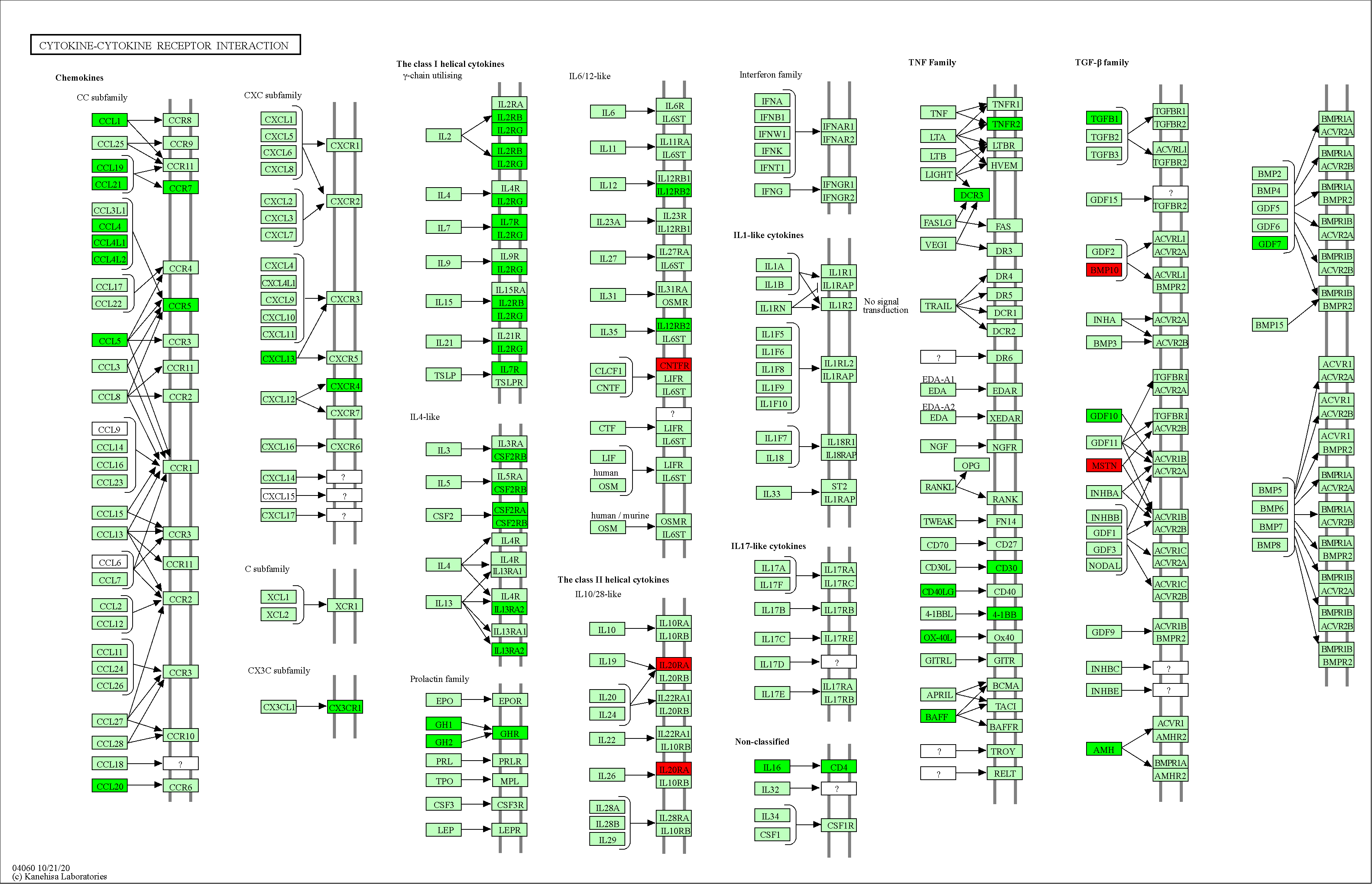


**Figure S1****9** DEGs in heart colored in the pathway of cytokine-cytokine receptor interaction.


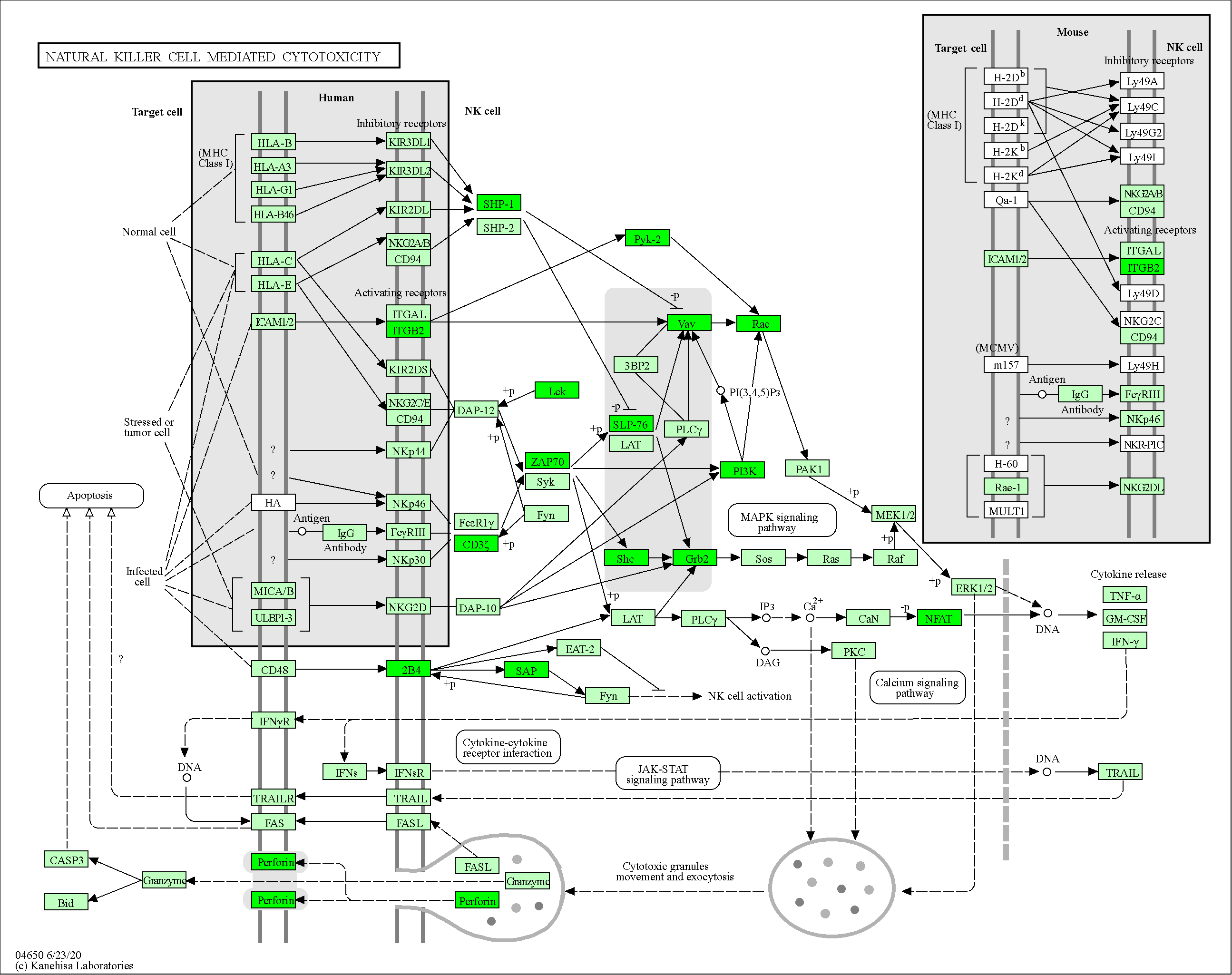


**Figure S****20** DEGs in heart colored in the pathway of natural killer cell mediated cytotoxicity.


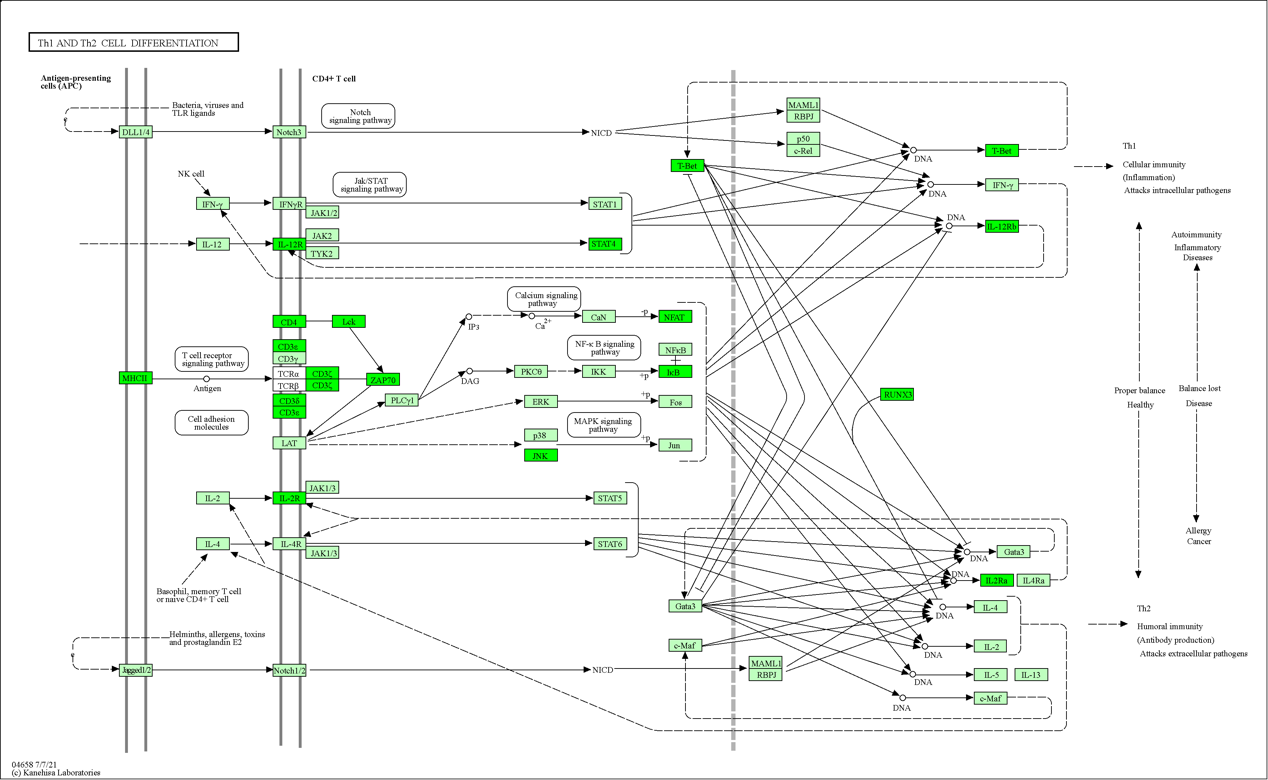


**Figure S****21** DEGs in heart colored in the pathway of Th1 and Th2 cell differentiation.


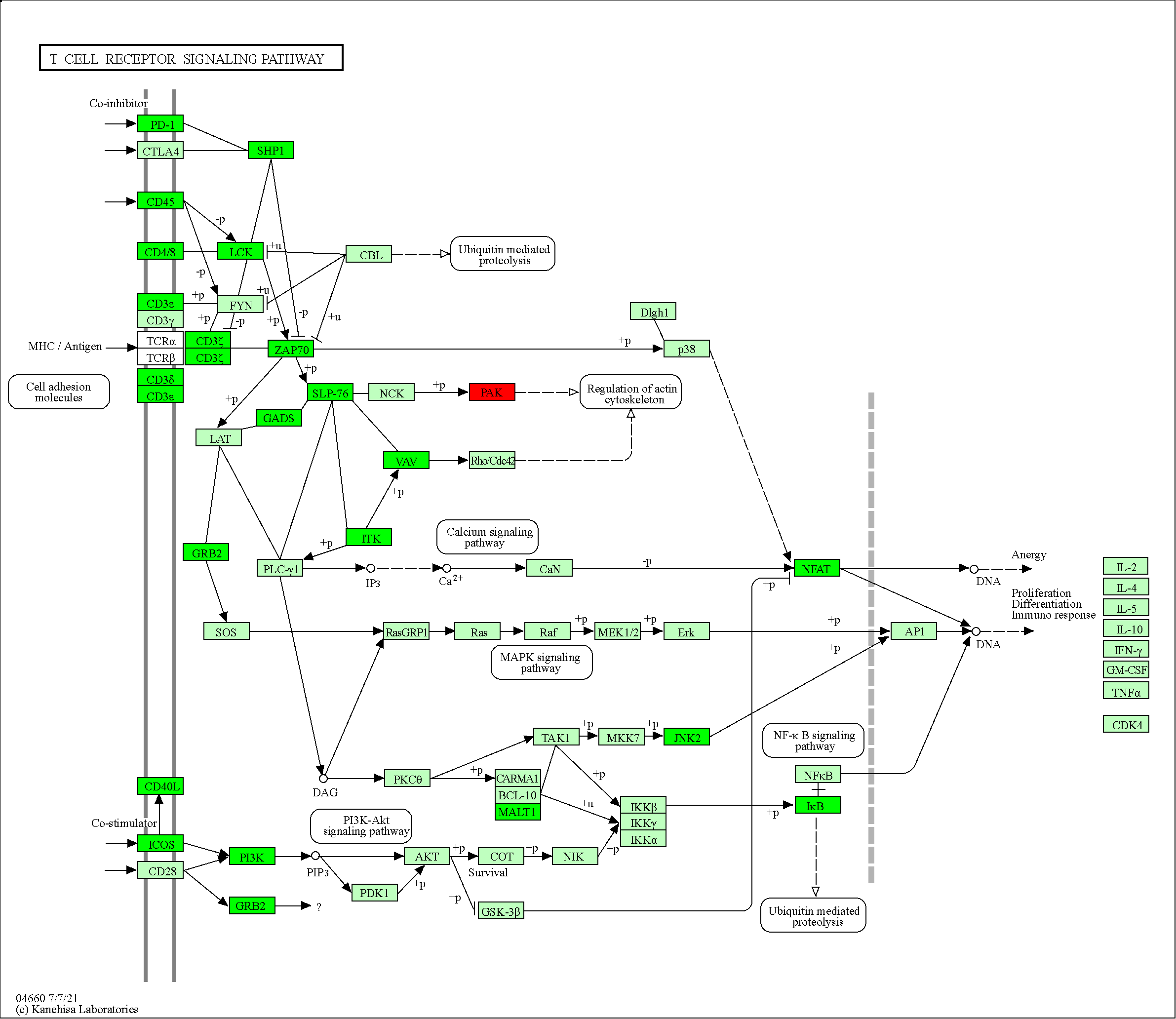


**Figure S22** DEGs in heart colored in the pathway of T cell receptor signaling pathway.


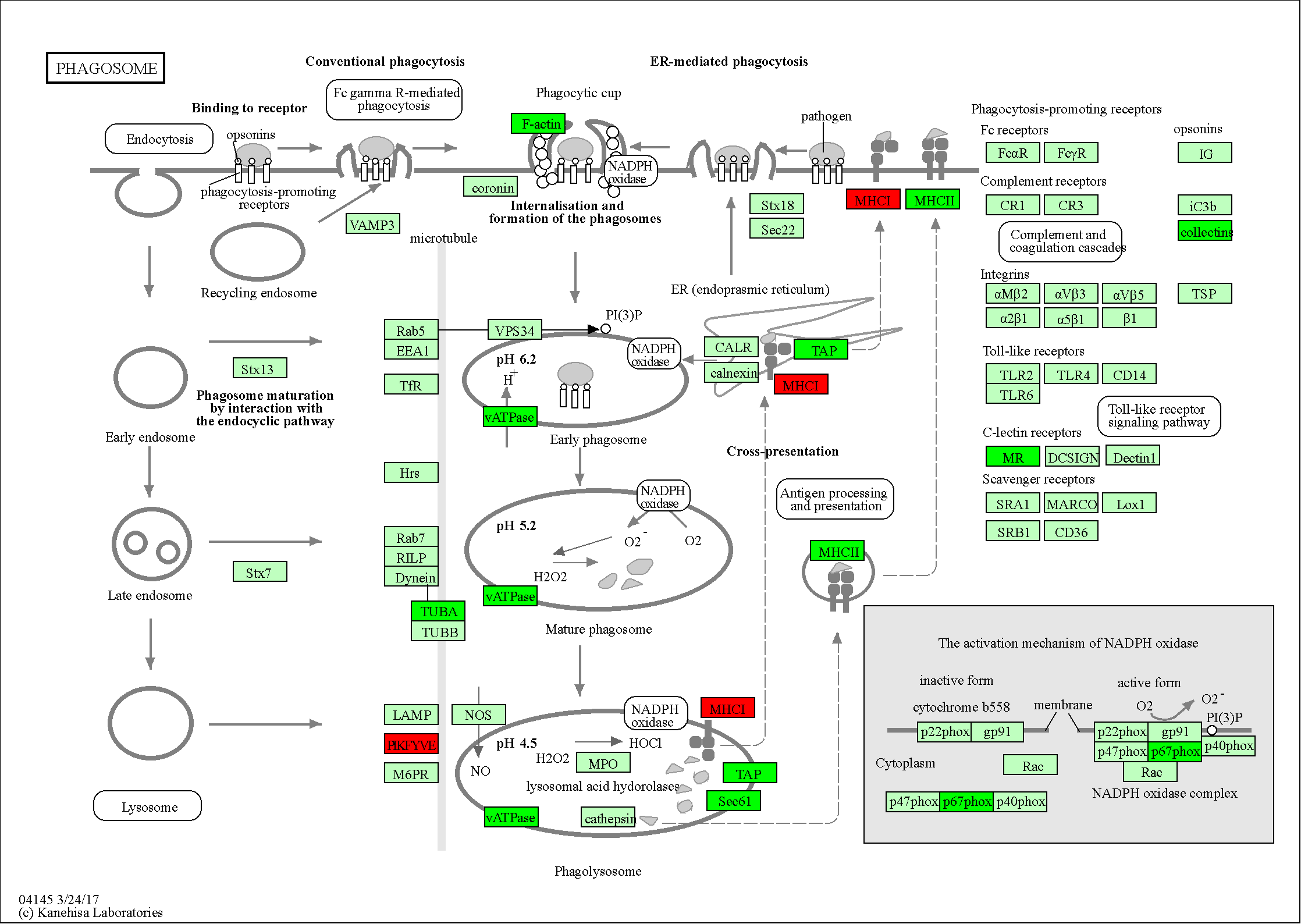


**Figure S23** DEGs in liver colored in the pathway of phagosome.
